# Experiences of running a stratified medicine adaptive platform trial: Challenges and lessons learned from 10 years of the FOCUS4 trial in metastatic colorectal cancer

**DOI:** 10.1177/17407745211069879

**Published:** 2022-01-27

**Authors:** Louise C Brown, Janet Graham, David Fisher, Richard Adams, Jenny Seligmann, Matthew Seymour, Richard Kaplan, Emma Yates, Mahesh Parmar, Susan D Richman, Philip Quirke, Rachel Butler, Kaikeen Shiu, Gary Middleton, Leslie Samuel, Richard H Wilson, Timothy S Maughan, On Behalf of the FOCUS4 Trial Investigators

**Affiliations:** 1 MRC Clinical Trials Unit at UCL, London, UK; 2 The Beatson West of Scotland Cancer Centre, Glasgow, UK; 3 Institute of Cancer Sciences, University of Glasgow, Glasgow, UK; 4 Centre for Trials Research, Cardiff University and Velindre NHS Trust, Cardiff, UK; 5 Leeds Institute of Medical Research at St James’s, University of Leeds, Leeds, UK; 6 Bristol Genetics Laboratory (BGL), Bristol, UK; 7 University College Hospital, London, UK; 8 University of Birmingham, Birmingham, UK; 9 Aberdeen Royal Infirmary, Aberdeen, UK; 10 MRC Oxford Institute for Radiation Oncology, Department of Oncology, University of Oxford, Oxford, UK

**Keywords:** Metastatic, colorectal cancer, biomarker, stratified, clinical trial, multi-arm multi-stage, adaptive, complex innovative design

## Abstract

**Background:**

Complex innovative design trials are becoming increasingly common and offer potential for improving patient outcomes in a faster time frame. FOCUS4 was the first molecularly stratified trial in metastatic colorectal cancer and it remains one of the first umbrella trial designs to be launched globally. Here, we aim to describe lessons learned from delivery of the trial over the last 10 years.

**Methods:**

FOCUS4 was a Phase II/III molecularly stratified umbrella trial testing the safety and efficacy of targeted therapies in metastatic colorectal cancer. It used adaptive statistical methodology to decide which sub-trial should close early, and new therapies were added as protocol amendments. Patients with newly diagnosed metastatic colorectal cancer were registered, and central laboratory testing was used to stratify their tumour into molecular subtypes. Following 16 weeks of first-line therapy, patients with stable or responding disease were eligible for randomisation into either a molecularly stratified sub-trial (FOCUS4-B, C or D) or non-stratified FOCUS4-N. The primary outcome for all studies was progression-free survival comparing the intervention with active monitoring/placebo. At the close of the trial, feedback was elicited from all investigators through surveys and interviews and consolidated into a series of recommendations and lessons learned for the delivery of similar future trials.

**Results:**

Between January 2014 and October 2020, 1434 patients were registered from 88 UK hospitals. Of the 20 drug combinations that were explored for inclusion in the platform trial, three molecularly targeted sub-trials were activated: FOCUS4-D (February 2014–March 2016) evaluated AZD8931 in the BRAF-PIK3CA-RAS wildtype subgroup; FOCUS4-B (February 2016–July 2018) evaluated aspirin in the PIK3CA mutant subgroup and FOCUS4-C (June 2017–October 2020) evaluated adavosertib in the RAS+TP53 double mutant subgroup. FOCUS4-N was active throughout and evaluated capecitabine monotherapy versus a treatment break. A total of 361 (25%) registered patients were randomised into a sub-trial. Feedback on the experiences of delivery of FOCUS4 could be grouped into three main areas of challenge: funding/infrastructure, biomarker testing procedures and trial design efficiencies within which 20 recommendations are summarised.

**Conclusion:**

Adaptive stratified medicine platform studies are feasible in common cancers but present challenges. Our stakeholder feedback has helped to inform how these trial designs can succeed and answer multiple questions efficiently, providing resource is adequate.

## Introduction

FOCUS4 is the first molecularly stratified platform trial to be undertaken in colorectal cancer and it remains one of the first umbrella trial designs to be launched globally.^1^ It is an example of a complex innovative design trial, and although these trials are becoming more common, they are still a rarity in the clinical research landscape. Recent high profile trials, such as the RECOVERY and PRINCIPLE trials,^2,3^ tested multiple treatments in the midst of the Covid-19 pandemic and generated practice-changing results swiftly and efficiently. They raised the visibility of these adaptive trial designs which have previously been implemented successfully over a number of years, for example, the STAMPEDE^4^ trial in prostate cancer. These trial designs offer great potential for testing treatments efficiently and improving outcomes for patients in a faster time frame^5,6^ and there is now a desire for increased use but they come with multiple challenges in terms of delivery and in some cases a lack of regulatory acceptance. Some of these challenges have been previously discussed in a number of research conduct publications with recommendations for improved uptake,^7–10^ but extending these experiences into the stratified medicine research arena has not yet been fully explored. We have already published a series of papers on the practical aspects of running these sorts of complex platform trials,^8–11^ but systematically eliciting learning experiences from all stakeholders within one large stratified medicine platform trial have not been reported previously and this is the primary objective of this article.

Terminology for innovative designs is broad and there is currently no consensus on how they should be named; so for the purposes of this article, we use the term platform trial to describe a variety of innovative designs, including umbrella designs, such as FOCUS4. Similarly, we use the term adaptive to describe FOCUS4 as it allowed trial arms to be added or dropped and also applied statistical stopping rules for the dropping of ineffective arms.

Critical to the success of any platform trial is the precise disease setting. The STAMPEDE trial in prostate cancer explores treatments for newly diagnosed advanced prostate cancer patients.^4,12,13^ The RECOVERY trial^2^ and PRINCIPLE trial^3^ address hospital- and community-based treatment of patients with Covid-19 but are not stratified. In FOCUS4, we selected the maintenance therapy setting following 16 weeks of first-line systemic therapy for metastatic colorectal cancer (mCRC).^1^ The rationale for this was built on prior data showing that intermittent chemotherapy was not inferior to continuous chemotherapy,^14,15^ and that this setting early in the disease course would provide the best opportunity for new treatment approaches to be tested before the onset of multiple resistance mechanisms which is typical of the more traditional approach in ‘last line’ settings.^1^

FOCUS4 was designed in 2011, funded in 2013 and launched in 2014. Our molecular understanding of colorectal cancer has dramatically increased over the last decade. It was in this context, prior to the further understanding of the complexity of colorectal cancer defined by the analysis of large cohorts of patients using RNA sequencing and bioinformatics clustering methods^16^ or single-cell RNA sequencing^17^ that FOCUS4 was designed. It has recently published the last of its results^18,19^ and this article describes our experiences of running such a complex trial over the 10 years since it was conceived, from the viewpoint of various key stakeholders.

## Methods

### The FOCUS4 design

**Figure 1.**
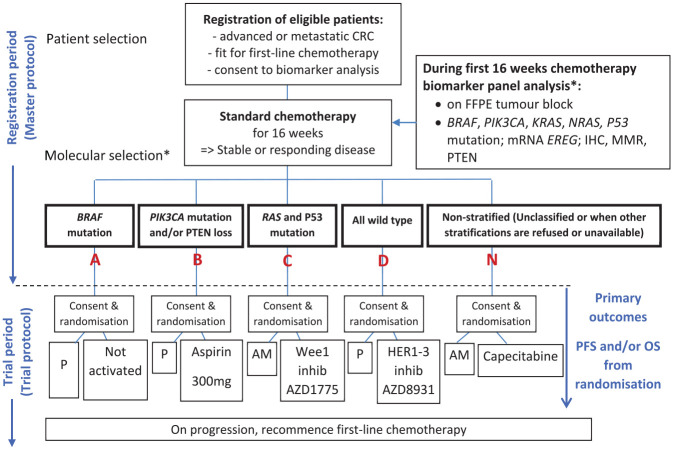
FOCUS4 trial schema. P: Placebo; AM: active monitoring; PFS: progression-free survival; OS: overall survival. *The molecular cohorts are arranged in a hierarchy from left to right. For example, a patient with both a BRAF and a PIK3CA mutation is classified into the BRAF mutation cohort.

**Figure 2.**
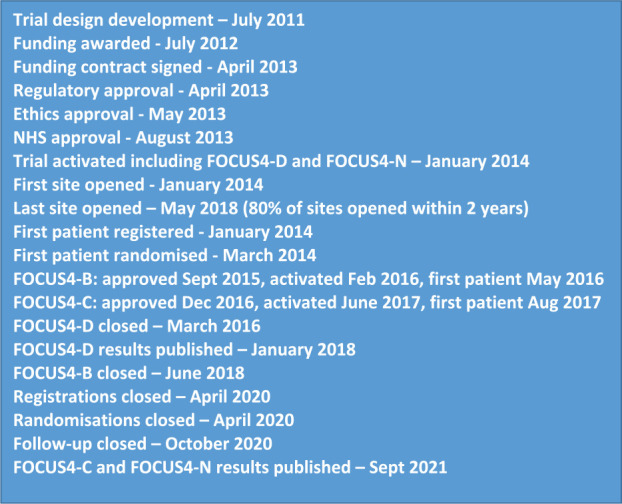
Timelines for the development and delivery of FOCUS4.

FOCUS4 was a Phase II/III molecularly stratified umbrella platform trial testing the safety and efficacy of targeted therapies in mCRC.^1^ The trial consisted of two periods: first, a registration period where tumour tissue was tested in all consenting patients; and second, a trial period where interventions were tested in a series of parallel molecularly stratified randomised controlled sub-trials and one non-stratified sub-trial. The trial schema is presented in Figure 1 with timelines in Figure 2. Full details on the trial methods and results have been published in the primary results papers for the sub-trials.^18–20^

In summary, patients with newly diagnosed mCRC were registered onto FOCUS4 while undergoing 16 weeks of first-line therapy. Following registration, a tumour sample was tested to stratify disease into molecular subtypes, including deficient mismatch repair, BRAF, PIK3CA, TP53 and RAS mutations. On completion of 16 weeks of first-line therapy, response was assessed by RECIST 1.1 reporting of pre- and post-therapy CT scans. Patients with stable or responding disease were eligible for randomisation into either a molecularly stratified sub-trial (FOCUS4-A, B, C or D) or into the non-stratified sub-trial (FOCUS4-N) which was offered to those in whom a targeted sub-trial was unavailable, who were unwilling to travel from their local hospital or in whom their biomarker tests failed. FOCUS4-N tested a generic research question comparing oral maintenance capecitabine therapy against active monitoring.

**Figure 3.**
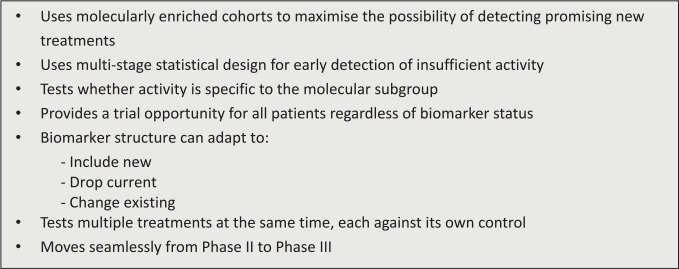
Key design features of FOCUS4.

Each molecularly stratified sub-trial started with a Phase II signal-seeking component that could proceed to a Phase III trial if the drug being tested demonstrated an adequate signal of activity at interim analysis. The Phase II primary outcome for all sub-trials was progression-free survival with the option to revise the trial sample size to explore overall survival as the primary outcome if the trial proceeded to Phase III. As part of this Phase III expansion, the design also allowed for the testing of biomarker specificity by evaluating the efficacy of the drug in a wider group of patients who were biomarker-negative. All these design features were integrated to improve the efficiency of testing multiple therapies within the same trial platform infrastructure with the key design features published previously^1^ and provided in Figure 3.

### Patient consent, registration and biomarker panel testing

Patients were approached to take part in FOCUS4 using a two-stage consent process. Initially, patient consent was obtained for registration and permission for biomarker testing of their tumour tissue and second when eligibility for a particular sub-trial had been established on the basis of the test results. Patient information sheets were provided for each stage of consent, and signed consent forms were required prior to registration or randomisation.

Patients were registered through an online registration platform managed at the Medical Research Council (MRC) Clinical Trials Unit (CTU) at UCL, and the movement of samples was tracked by the MRC CTU from the local site pathology departments to one of the two mutually quality assured laboratories in Cardiff (Department of Cellular Pathology and All Wales Molecular Genomics Laboratory, Institute of Molecular Genetics, both located at University Hospital of Wales) and Leeds (Division of Pathology and Data Analytics, Leeds Institute of Medical Research at St James’s, University of Leeds). Laboratory testing initially comprised pyrosequencing of the mutation hotspots and from August 2017 using whole gene next-generation sequencing, plus immunohistochemistry for mismatch repair proteins and phosphatase tensin homologue (PTEN). The technical components of the biomarkers and inter-laboratory quality assurance have been described previously.^21^ For patients in whom their fixed paraffin embedded (FFPE) tumour blocks did not contain sufficient tumour tissue or the tests failed, the patient was still eligible for entry into the FOCUS4-N sub-trial.

### Participating sites

A total of 103 hospitals were activated in FOCUS4 across all four devolved UK nations. All sites were able to register patients into FOCUS4 but given that the drugs being tested in the randomised comparisons ranged from novel unlicensed drugs to generic therapies; sites were assessed for relevant capacity and expertise for participation in each of the comparisons. Sites were classified into three levels.

*Level 1 sites (n* *=* *51)*: Hospitals with clinical trial experience but without the required expertise for testing unlicensed therapies. Level 1 sites could register patients and recruit into FOCUS4-B (testing aspirin) and FOCUS4-N (testing capecitabine).
*Level 2 sites (n* *=* *29)*: Hospitals with experience of testing both licensed and unlicensed drugs but without extensive early phase experience. Level 2 sites could register patients and randomise into FOCUS4-B, FOCUS4-D, FOCUS4-N and eventually into FOCUS4-C when safety and tolerability of the Wee-1 inhibitor had been assessed by the Independent Data Monitoring Committee.
*Level 3 sites (n* *=* *23)*: Hospitals with early phase experience and extensive clinical trials experience of licensed and unlicensed drugs. Level 3 sites could register and randomise into all comparisons.

### Statistical methods

FOCUS4 was designed to allow randomised sub-trials to be added into the platform as new agents became available or drop agents if pre-specified interim analyses indicated a lack of sufficient drug activity. The inclusion of new agents required peer-review by a joint sub-board meeting of the two funders (National Institute for Health Research (NIHR) Efficacy and Mechanism Evaluation (EME) and Cancer Research UK (CRUK)). Decisions on the stopping of particular sub-trials were based on multi-arm multi-stage statistical methodology^5,22–25^ such that the Independent Data Monitoring Committee was provided with pre-specified stopping guidelines for each sub-trial and asked to review the data in confidence at interim analyses and make recommendations on whether to continue or stop the sub-trial. These recommendations were considered by the Trial Steering Committee and Trial Management Group without sight of any data before a stop/go decision was made for that sub-trial. Full details on the statistical methods, including sample size calculations, stopping rules and analytical methods, are provided in the primary results papers.^18–20^

#### Activation and recruitment

Timelines for FOCUS4 are provided in Figure 2. FOCUS4 was co-funded by the MRC/NIHR EME Programme and CRUK with funding commencing in April 2013. The trial was approved by the UK National Ethics Committee Oxford – Panel C (reference 13/SC/0111) and by the relevant regulatory body Medicines and Healthcare products Regulatory Agency (MHRA; CTA# 20363/0400/001 and EudraCT# 2012-005111-12) in May 2013. FOCUS4 opened to recruitment in January 2014 with two sub-trials activated (FOCUS4-D and FOCUS4-N). Over the following 6 years, 103 UK sites were opened and subsequent substantial amendments approved the inclusion of two further sub-trials (FOCUS4-B and FOCUS4-C). Activating the initial 103 sites took over 4 years with ∼80% activated in 2 years. However, activating each new sub-trial (FOCUS4-B and C) as substantial amendments took approximately 7 months (see Figure 2).

Considerable challenges were encountered engaging pharmaceutical companies to test therapies in the platform with 20 drug combinations explored for inclusion. Of the five sub-trials that we had planned to open at the design stage (A, B, C, D and N), only four were activated (B, C, D and N) and three reported results (C, D and N) with FOCUS4-B stopping early on grounds of futility.

In terms of project funding, typically a standard two-arm trial would have budgeted for one trial manager and one data manager for the duration of the trial along with ∼25% of a statistician and 25% of a data scientist for analysis and database activities. For FOCUS4, the final grant budgeted for 1.5 trial managers and 1.5 data managers. In reality, we used at least two trial managers and two data managers throughout the trial with some temporary additional support needed from core staff at times of intense activity. We needed far more database programmer time than we had budgeted for as the database needed full testing each time a new comparison was added. We eventually decided to separate the FOCUS4 database into a modular form so that future changes in one sub-trial did not require testing across the whole database. All these learning points have been described in our various other operational publications.^8,9^ Similarly, we required more statistical time than we had budgeted for with %FTE varying between 25% and 50% from year to year when we actually needed 50% of the statistician working on the trial most of the time.

The trial is registered at https://www.isrctn.com/ (ISRCTN# 90061546). Full information, including the trial protocol, can be found at the FOCUS4 website: http://www.focus4trial.org/.

### Failed endeavours

A key drain on investigator time was the continual pursuit of new agents for testing in the FOCUS4 platform. During the course of the trial, a total of 20 drug combinations were explored and presented to the joint NIHR EME/CRUK funding sub-board for peer-review approval but only 4 culminated in an activated comparison. Table 1 summarises these attempts with reasons for non-activation predominantly being due to the failure of drugs at early clinical testing in this advanced metastatic disease setting when protocol development and contract negotiations had often progressed a long way and considerable resource had been used up for ultimately futile collaborations. Other endeavours failed due to strategic shifts within companies, including the selling of assets suddenly and unexpectedly.

**Table 1.** Summary of 4 successful and 16 unsuccessful comparisons explored for addition to the FOCUS4 platform.

Cohort	Biomarker	BMincidence (%)	Intervention	Outcome
A1	BRAF V600E mutation	10	BRAF I and MEKi	Science evolved
A2	BRAF V600E mutation	10	Dabrafenib, trametinib+panitumumab	GSK sold oncology portfolioto Novartis. Novartis: insufficient activityto support.
B1	PIK3CA mutant or PTEN loss on IHC	22	Dual PI3Ki/mTORi	Insufficient evidence of benefit
B2	PIK3CA mutation	12	Aspirin	FOCUS4-B trial
C1	KRAS/NRAS mutation	45	MEKi + PI3Ki	Found to be too toxic inearly studies
C2	RAS mutation + HLA A-2	20	IMA 190 peptide vaccine	Company did not commit
C3A	H3K36me3 loss	<2	Wee-1 inhibitor AZD1775	Biomarker: very low incidenceof loss
C3B	RAS + TP53 double mutation	30	Wee-1 inhibitor	FOCUS4-C trial
C3C	ATM loss	6	ATM inhibitor AZD 6738	Company did not support concept
D1	KRAS, NRAS and BRAF wildtype	40	Pan-HER inhibitor AZD8931	FOCUS4-D trial
D2	KRAS, NRAS and BRAF wildtype	40	MM151	Company sold asset prior tocontract
D3	Triple wildtype, HER2 negative	25	Cetuximab + CDK4/6i	Pending data from SCCHN
E1	MMR deficient and POLE mutant	4	Avelumab	Company did not support concept
E2	MMR deficient or TGFb activated	30	Bintrafusp-alfa	EME/CRUK did not extend grant
F	Axin 2 overexpressed	9	RXC004 porcupine inhibitor	EME/CRUK did not extend grant
G	HER-2 overexpressed	2	Trastuzumab + CDK4/6i	Biomarker incidence too low
H	ALK/ROS rearrangements	2	Crizotinib	Biomarker incidence too low
N1	Non-stratified group	-	Capecitabine	FOCUS4-N trial
N2	Non-stratified group	-	Add TAS-102	Global company did not support concept
N3	Non-stratified group	-	Metronomic cyclophosphamide	EME/CRUK did not extend grant

### Reflections from stakeholder groups involved in delivery of the FOCUS4 trial

With FOCUS4 now closed and to learn more from our 10-year experience and provide insight for those in the research community hoping to undertake similar studies, we sought feedback from a number of stakeholders who were involved in the delivery of the trial. Each of the following eight stakeholder groups were asked to provide learning points from their experience: (1) The co-chief investigators, (2) sub-trial chief investigators on the Trial Management Group, (3) clinical research fellow trainees, (4) the MRC Clinical Trials Unit, (5) the biomarker testing laboratories, (6) patient/carer representatives, (7) oversight committees and (8) funders. The request for feedback was free and unstructured with any comments welcomed.

In addition, a participating site survey was distributed through site principal investigators to understand the experiences of site staff. Principal investigators at each of the 88 sites who registered at least one patient were sent a link to an anonymised survey through survey monkey and asked a series of questions about their practice and encouraged to provide additional comments on the aspects of the trial that had worked well and not so well. In addition to feedback on trial-specific processes, the survey asked specifically about sites experiences of accessing drugs, such as eGFR inhibitors within the UK National Health Service, to see if rulings on access had any impact on willingness to register or randomise patients. See the Supplemental material Appendix 2 for a copy of the survey.

## Results

### Results from FOCUS4 trial

Full details on the results for the registration and sub-trial phases of FOCUS4 have been published in the primary results papers for the sub-trials.^18–20^

Between January 2014 and March 2020, 1434 patients were registered from 88 of 103 UK participating hospitals and 361 were randomised into a sub-trial (25%). Biomarker analysis was undertaken on 1382 samples, and the median time from registration to availability of the biomarker results was 6 weeks (interquartile range 4.3–8.3 weeks). Molecular stratification was successful in 1291/1382 (93%) of the samples tested. Of the 1315 patients completing 16 weeks of first-line therapy, 908 (68%) had disease stabilisation or response and were potentially eligible for randomisation. Of these, 361 were randomly allocated in one of the sub-trials: FOCUS4-B (*N* = 6); FOCUS4-C (*N* = 69); FOCUS4-D (*N* = 32) and FOCUS4-N (*N* = 254). FOCUS4-B closed recruitment in 2018 due to poor recruitment, FOCUS4-D closed in March 2016 following a lack-activity interim analysis and reported results in 2018^20^ and FOCUS4-C and N reported in 2021^18,19^ with positive results in both trials. The whole FOCUS4 platform closed in October 2020 to enable final reporting before grant funding ended in 2021 (see Figure 2 for timelines).

### Feedback from stakeholder groups

We received feedback from 19/38 individuals including at least one representative from each of the eight stakeholder groups. Experiences were very positive despite an acknowledgement of the substantial challenges encountered. There was considerable overlap in a number of the points raised and the comments naturally grouped into three main areas: (1) resource and infrastructure, (2) biomarker testing process and (3) trial design. From these, we synthesised 20 learning points that are summarised in Table 2.

**Table 2.** Key learning points from stakeholder feedback.

	Resource and infrastructure
1	Secure adequate funding
2	Delivering all desired outcomes for a platform trial is clearly challenging. The challenge for funders is to find a mechanism for funding and reviewing of adaptations that facilitates delivery and minimises burden while also managing the risks involved.
3	Ideally, these trials should only be conducted in clinical trial units that have good core funding resources and a ballast of trained in-house trial and data managers who can be drawn upon temporarily at times of intense activity.
4	Activate fewer sites and stagger opening.
5	Leadership: The Chief Investigator (CI) role is paramount and must not be underestimated with far more pressure than being a CI on a more standard trial. An engaged and enthusiastic core Trial Management Group (TMG) is vital.
6	The Clinical Trials Unit (CTU) staff must feel comfortable and encouraged to escalate any site issues to senior TMG members quickly.
7	A great training experience for CTU staff and clinical research fellows. Provide basic clinical trial training for research fellows to aid learning.
8	Site enthusiasm was inconsistent between registration and randomisation. Understand local motivations or obstacles to recruitment.
9	Trial longevity can lead to poor continuity of CTU and site staff which is disruptive in a complex trial where the design keeps adapting.
10	For trials that last many years, trial participants need better opt in/opt out arrangements on how they can be kept informed on trial progress.
	Biomarker testing process
11	Regular quality assurance (QA) and review of sample testing processes to identify any glitches that require modification.
12	Important to spend adequate time on biomarker workup and optimisation, and understanding prevalence early on before taking further.
13	Keep biomarker testing within the NHS infrastructure as much as possible with as few middle men as possible to avoid data privacy obstacles.
14	Important to have an engaged and dedicated biomarker team who can manually step in and overcome any delays to prevent patient distress.
	Trial design
15	The multi-arm multi-stage (MAMS) adaptive design worked well at cutting losses on poorly performing drugs early.
16	The requirement for a control arm in each comparison was important in determining any prognostic biomarker effects.
17	The need for a catch-all non-stratified trial (FOCUS4-N) proved to be successful at maximising trial opportunities for patients.
18	Important to get the protocol structure right and consult with regulatory bodies on advice for what is acceptable within the design.
19	The main issues were mainly related to pharma engagement and drug-target identification in the specific disease setting of the study. Earlier engagement in the developmental pathway for new therapies is required so that when the therapy is ready to drop into a trial, all parties have been engaged and involved with the biomarker optimisation and early drug activity assessments.
20	Funding of complementary feeder collaborations, such as SCORT (which focused on understanding the biology) and ACRCelerate (which focussed on pre-clinical novel agent development), might have been beneficial if run in parallel with FOCUS4.

The most consistent learning points related to five key areas:

1. Understand resource capacity and ensure that adequate funding is secured for staff. These designs probably save time and speed up getting answers but they still require similar amounts of resource per research question. The challenge for funders is to find a mechanism for funding and review of trial adaptations that facilitates delivery and minimises burden while also managing the risks involved.
2. The biomarker testing process should be kept as simple as possible and for the United Kingdom, as much as possible within the National Health Service infrastructure.
3. Position the trial within the optimal phases of drug development and try to ensure there are parallel research initiatives to support the trial in relation to ever-shifting biology and pre-clinical workup. FOCUS4 may have worked better as a Phase I/IIb platform. Had biomarker and drug combinations been further developed, the Phase II/III design might have worked better.
4. Platform trials need to be nimble and able to adapt quickly with emerging new biological discoveries. This is difficult in a sometimes turgid clinical trial regulatory framework.
5. Engagement, tenacity and enthusiasm are paramount from the Chief Investigator and Trial Management Group. Without this, a trial of this complexity would fail.

### Results from the site survey

We received feedback from 52/88 sites (59%) with representation from all 1, 2 and 3 level sites, but we chose not to compare responses between the levels of sites as numbers were small. Sites were asked about standard treatment for patients with unresectable Stage 4 mCRC; the majority either used a block of chemotherapy followed by a complete break or intermittent chemotherapy with a complete break. In some settings, treatment to progression was considered and also maintenance chemotherapy, but both were rare. Most sites were supportive of using the maintenance setting to test new therapies. As with many trials, recruitment rates varied substantially between sites and much of this variation related to the level of engagement from the site principal investigator and the local processes and prioritisation of recruiting cancer patients into academic trials.

The remaining results of the survey have been summarised in Table 3 where it is clear that although sites experienced a number of challenges in delivering FOCUS4, they were positive about their involvement and supportive of the use of these trials in the future, if resourced adequately. For many sites, the main obstacle was not the operational aspects of the trial but the lack of exciting new therapies in the platform and in the mCRC research space in general.

**Table 3.** Summary of results from participating site survey.

Question and response (%)	Take home message
***Did inability to restart an EGFRi impact on patient selection?*** Agree/ strongly agree – 68% Neutral – 14% Disagree/ strongly disagree – 18%	NHS rulings on the use of eGFR inhibitors restricted recruitment and may have been a barrier to finding alternative or better therapies relevant in the RAS wildtype group.
***Was having an unselected FOCUS4-Ntrial important?*** Agree/ strongly agree – 71% Neutral – 25% Disagree/ strongly disagree – 4%	An important aspect of the design that was strongly supported by sites and patients
***What were the advantages anddisadvantages of conducting this trial in the maintenance setting?***	**Advantages:** •Fitter patients •Less acquired drug resistance •Induction chemo allowed time for biomarker testing without delaying treatment start •Less end organ impairment •Patients felt they were ‘trying something’ when otherwise might be having a break **Disadvantages:** •NHS England rules preventing EGFRi reintroduction •A more challenging route to registration for successful agents •Additional hospital time or toxicity •Some patients progress during induction treatment and become ineligible
***Did you experience any particular study challenges?***	**Staff and infrastructure** •Delays with local Research & Development (R&D) department approval •Limited nursing support particularly at Level 1 sites for our network •Maintaining team motivation when novel arms not open •Some challenges referring from Level 1 to 2 or 3 site. **Trial assessments** •Poor capacity for RECIST reporting •Novelty of the trial biomarker panel became diluted as NHS testing rolled out.
***What went well?***	•Excellent CTU communications (response to queries, newsletters, etc.) •Novel design of an adaptive platform trial in a common solid tumour – first of its type in the United Kingdom •Easy to recruit with the window to request biomarker testing •Engaged all geographical areas within the United Kingdom with the Levels 1, 2, 3 designs. •Patient information sheets were well developed.
***Are platform trials the future?***	•Grossly underfunded but definitely the best way to proceed compared to running endless small trials in small subgroups •Funders should have supported funding for fresh biopsies and additional translational work •Speed and efficiency of adding arms with protocol amendments •Platform allows for sub-studies, for example, exercise, PET, CtDNA.

## Discussion and conclusion

Adaptive trials not only provide significant advantages in the evaluation of multiple novel therapies in a disease setting but also provide major challenges in their design, funding and delivery. FOCUS4 was jointly funded by the MRC/NIHR EME Programme and CRUK with a combined budget of £3.6M, which was large in 2012 but small in comparison to current major molecularly stratified platform studies. Joint funding added a delay to trial initiation and review of amendments (performed through a sub-board representing both funders). Early on in the trial, it became apparent that the staff usage costed into our original budget was inadequate and additional financial support was required throughout the entire trial. For these trials to fulfil their aim of flexibility and nimbleness, we need to learn and implement the lessons of undertaking complex trials in the era of the pandemic, particularly in terms of protocol and amendment approval as recently espoused in the United Kingdom.^6^ The efficiencies of FOCUS4 are evident in Figure 2, where activation of original sites took many years while implementation of new sub-trials as amendments took a matter of months.

The key features outlined in our design paper (see Figure 3) have all proved to be robust in the application. The use of progression-free survival in the maintenance setting as the primary endpoint has been shown to be an effective indicator of agents with activity, which may not have been revealed through a more conventional assessment of response in end stage patients. This is of particular relevance for trials in the maintenance therapy setting. We were not able to take a therapy as planned through to test whether the activity is specific to the molecular subgroup initially identified or to move from Phase II to Phase III testing, but this was due to timing and funding availability rather than design failure.

The setting of the interval after 16 weeks of induction chemotherapy for patients with metastatic disease is a novel one. The recent licensing in this setting of olaparib in BRCA mutant cancer has made this a more recognisable route to registration. It builds on a pattern of practice of allowing patients a complete break from therapy following several months of induction chemotherapy which is well evidence-based but while not unique to the United Kingdom is perhaps more widely utilised in the United Kingdom than many other countries. This may change as a result of the Covid-19 pandemic as patients now choose to spend more time away from hospitals and many have preferred a move to remote monitoring.

Adding the complexity of molecular stratification to allocate patients into the optimal biomarker defined subgroup adds an extra layer of challenges including the developmental status of the biomarker, its predictive power and selectivity for the therapy being evaluated (which is usually underdeveloped for any novel agent). Timely and reproducible delivery of the biomarker testing panel, understanding the prognostic impact of the biomarker selection and the need to accommodate biomarker-negative patients remains critical to success. During FOCUS4, the simple mutation-based stratification process for many of the biomarkers we tested has become part of standard care. In the future, despite the now more widespread routine availability of next-generation sequencing-based tumour profiling, trial-based stratification testing will continue to need to provide transcriptomic and other more sophisticated analyses. It may be that the revolution in digital pathology and artificial intelligence will enable some biological stratification to be achieved directly from routine images as shown recently with consensus molecular subtyping.^26^

In undertaking FOCUS4 as a platform design, we have noted some important distinctions between a stratified platform study and a stratified stand-alone study. Optimising the biomarker testing process and completing the necessary quality assurance between testing centres are an important part of any stratified trial activation. This took at least a year for FOCUS4 but was happening in parallel with other trial set-up activities. This meant that CTU staff were taken away from other generic set-up tasks. This would be the same for a stand-alone stratified medicine trial but perhaps only one or two biomarkers would be measured while multiple sub-trials in a platform require a panel of results that all have to undergo quality assurance. Regular quality assurance review is needed throughout the trial and this is likely to be more of an issue for platform stratified trials as they go on for longer and need to incorporate new biomarkers and updated technology as new sub-trials are added to the platform. For any stratified medicine study, it is important that there are dedicated CTU staff to handle the sample tracking process. We found that this required one 100% FTE member of staff at the CTU with varying %FTE staff at each testing lab. This may be about the same as a stand-alone stratified medicine study, but the efficiency is that more patients are being used for multiple sub-trials rather than just selecting patients with one particular biomarker and excluding the rest. Processes would have been far simpler if our panel of biomarkers could have been measured routinely as part of standard care at each site. However, novel biomarkers are likely to always be needed for earlier Phase II studies, such as FOCUS4, where new biomarkers are being tested with particular targeted therapies.

Rates of allocation into biomarker-selected groups are often very low in precision medicine studies as exemplified by the Lung-Matrix Trial^27^ and the Lung-Map Trial.^28,29^ In the largest study of this kind, NCI-MATCH,^30^ 5954 patients were enrolled with refractory malignancies, of whom 17.8% were assigned to a targeted therapy. Among these, 848 colorectal cancers were registered, 13.7% of whom were assigned and 10% enrolled into a specific therapy trial.^31^ In FOCUS4, rather than assigning patients in non-randomised cohorts to therapies with hypothesised efficacy, we randomised 361/1434 (25%) of registered patients into a specified randomised sub-trial, and 107 (7.5%) of registered patients into molecularly stratified sub-trials. These numbers are remarkably comparable to those seen in the Lung-Map trial^29^ which has an almost identical umbrella design. In Lung-Map, although a higher proportion of all 1864 registered patients entered any sub-study (35%), only 11% entered a biomarker sub-study. The reasons for attrition in FOCUS4 were also similar to those for the Lung-Map trial and are outlined in more detail in our primary results publications.^18,19^ In summary, they related to three main factors: inadequacy of sample submission or analysis (10%), death or progressive disease during induction therapy (27%) or lack of availability of a suitable molecular sub-trial or lack of patient consent when the patient was eligible for randomisation (60% of those potentially eligible).

It is notable from the RECOVERY trial, which tested multiple treatments in a non-stratified design, that negative results are more likely but are just as important as positive results in tackling diseases of high unmet clinical need. To date, the RECOVERY trial has identified two positive results (dexamethasone and tocilizumab) and four negative results in the 1 year since its set up in March 2020. NCI-MATCH^32^ has reported seven cohorts to date of which three have shown positive outcomes. For Lung-Map, nine sub-studies were activated with one demonstrating a positive outcome, six closing early due to futility and the other two closing for reasons external to the study. In FOCUS4, we report two positive results (capecitabine and adavosertib), one clear negative (Her 1, 2, 3 inhibition) and one feasibility failure (aspirin).

The importance of adding a non-stratified research question into the platform (FOCUS4-N) was a clear strength of the design, one that was also exemplified in the US Lung-Map trial^29^ where the ratio of patients entering non-stratified to stratified sub-studies was approximately 2:1, as seen in FOCUS4. To maximise efficiency from future trials, we would advocate answering multiple research questions across different biomarker groups in one trial that also includes a non-stratified generic research question. Thus, the use of platform trials in stratified medicine is one way in which we can understand diseases better while also maximising trial opportunities for patients, regardless of their biomarker status.

While non-commercial organisations may be best placed to set up and run such studies enabling collaboration with differing pharmaceutical companies for different agents, engagement with Pharma is critical to success. A large amount of investigator time was spent negotiating with companies to test their agents in the platform but only 4 out of 20 drug combinations explored came to fruition. To obtain approval from companies to include their agents, a very robust approach to the development of pre-clinical data packages to support selection of particular drug/biomarker combinations is essential. This requires a well-funded, pre-clinical testing collaboration ideally with disease subtype-specific models using GEMMs, PDX and PDOs linked to detailed disease stratification information. From this basis, strong applications can be made to Pharma to include agents in such precision medicine studies with a higher likelihood of success than we observed in FOCUS4. Only in the last 2 years has this been available through separate funding streams (the MRC stratified medicine consortium S: CORT and the ACCelerate collaboration faintly funded by CRUK, AIRC and FC AECC) and the fruit of this is yet to be seen to feed into an updated precision medicine study in CRC.

The enterprise of precision medicine adaptive platform trials is a massive exercise in team science and we hope that the lessons we have learned and summarised in Table 2 are helpful to others in embarking on such trials. It is a tribute to the large body of investigators, research nurses and data managers at sites (see Supplemental materials), laboratory scientists, trials unit staff with support from the institutions, funders and pharmaceutical companies and central to all this our patients which have enabled us to complete this FOCUS4 trial.

## Supplemental Material

sj-docx-1-ctj-10.1177_17407745211069879 – Supplemental material for Experiences of running a stratified medicine adaptive platform trial: Challenges and lessons learned from 10 years of the FOCUS4 trial in metastatic colorectal cancer Supplemental material, sj-docx-1-ctj-10.1177_17407745211069879 for Experiences of running a stratified medicine adaptive platform trial: Challenges and lessons learned from 10 years of the FOCUS4 trial in metastatic colorectal cancer by Louise C Brown, Janet Graham, David Fisher, Richard Adams, Jenny Seligmann, Matthew Seymour, Richard Kaplan, Emma Yates, Mahesh Parmar, Susan D Richman, Philip Quirke, Rachel Butler, Kaikeen Shiu, Gary Middleton, Leslie Samuel, Richard H Wilson and Timothy S Maughan in Clinical Trials

sj-pdf-2-ctj-10.1177_17407745211069879 – Supplemental material for Experiences of running a stratified medicine adaptive platform trial: Challenges and lessons learned from 10 years of the FOCUS4 trial in metastatic colorectal cancer Supplemental material, sj-pdf-2-ctj-10.1177_17407745211069879 for Experiences of running a stratified medicine adaptive platform trial: Challenges and lessons learned from 10 years of the FOCUS4 trial in metastatic colorectal cancer by Louise C Brown, Janet Graham, David Fisher, Richard Adams, Jenny Seligmann, Matthew Seymour, Richard Kaplan, Emma Yates, Mahesh Parmar, Susan D Richman, Philip Quirke, Rachel Butler, Kaikeen Shiu, Gary Middleton, Leslie Samuel, Richard H Wilson and Timothy S Maughan in Clinical Trials
