# Supplementary material for: Experiences of running a stratified medicine adaptive platform trial: Challenges and lessons learned from 10 years of the FOCUS4 trial in metastatic colorectal cancer
Source: Clin Trials. 2022 Jan 27;19(2):146–57. doi: 10.1177/17407745211069879 (PMC9036145; doi:10.1177/17407745211069879)
Supplement: sj-docx-1-ctj-10.1177_17407745211069879 – Supplemental material for Experiences of running a stratified medicine adaptive platform trial: Challenges and lessons learned from 10 years of the FOCUS4 trial in metastatic colorectal cancer [file sj-docx-1-ctj-10.1177_17407745211069879.docx]

**Full list of FOCUS4 trial investigators**

Writing committee:

Brown LC, Graham J, Fisher D, Adams R, Seligmann J, Seymour M, Kaplan R, Yates E, Parmar M, Richman SD, Quirke P, Butler R, Shiu K, Middleton G, Samuel L, Wilson RH, Maughan TS.

Trial Management Group:

Maughan TS (Chair), Wilson RH, Adams R, Seymour M, Seligmann JF, Graham J, Wasan H, Pope M, Pope J, Samuel L, Shiu K, Church D, Middleton G, Steward W, Twelves C, Wellman S, Hodgkinson E, Stoner N, Beety J, Duggleby K, Dutton G, MRC CTU team (see below), Laboratory staff (see below).

MRC CTU trial coordinating centre:

Brown LC, Kaplan R, Parmar M, Fisher D, Campos M, Yates E, Santana S, Fiddament A, Harper L, Bara A, Pugh C, Bathia R, Letchemanan K, Przybył B, Bhogal S, Gopalakrishnan G, Purvis C, Diaz-Montana C, Mohamed F, Townsend S, Cragg W, Masters L, Van Looy N, Rauchenberger M.

Laboratories:

Quirke P, Richman SD, Hemmings G, Davis J, Gallop N, Wilkinson L, Butler R, Roberts H, Jasani B, White R, Dodds R, James M, Morgan M.

Independent Data Monitoring Committee:

Cameron D (Chair), Souhami R, Peeters M, Billingham C, Griffiths G, Brown J

Trial Steering Committee:

Johnson P (Chair), Rudd R, Whelan J, Russell A

Participating hospitals in descending order of number of patients registered with all staff listed (N=2076)

| **Hospital** | **First name** | **Surname**  **PI (Principal Investigator)** |
| --- | --- | --- |
| **Western General Hospital** | Ewan | Brown (PI) |
|  | Morven | Atkinson |
|  | Caroline | Bruce |
|  | Patricia | Campbell |
|  | Sally | Clive |
|  | Kathryn | Connolly |
|  | Lorraine | Cook |
|  | Helen | Creedon |
|  | Judith | Crawford |
|  | Alisa | Davinson |
|  | Lesley | Dawson |
|  | Grace | Ding (Tze-en Ding) |
|  | Martin | Doak |
|  | Tamasin | Doig |
|  | Clare | Dunbar |
|  | Ben | Elliott |
|  | Lynne | Faragher |
|  | Paul | Fineron |
|  | Nikki | Gilluley |
|  | Ewa | Kondarewicz |
|  | Jenifer | Lauchlan |
|  | Jim | Macpherson |
|  | Catriona | Mclean |
|  | Hazel | Milligan |
|  | Suzanne | Muir |
|  | Alisa | Oswald |
|  | Ashley | Pheely |
|  | Hamish | Phillips |
|  | Kelly | Rust |
|  | Shaafia | Siddiqui |
|  | Barbara | Stanley |
|  | Moira | Stewart |
|  | Vivienne | Wilson |
| **St James University Hospital (Leeds)** | Fiona | Collinson (PI) |
|  | Matt | Seymour (PI) |
|  | Humaira | Abbas |
|  | Ifeoluwa | Ajibayo |
|  | Alan | Anthoney |
|  | Hana | Ali |
|  | Orrie | Appell |
|  | Andrew | Barker |
|  | Vincent | Barlow |
|  | Shanaz | Begum |
|  | Maxine | Berry |
|  | Judith | Chapman |
|  | Sam | Charlton |
|  | Pam | Clark |
|  | Emily Rose | Crawford |
|  | Anne | Crossley |
|  | Gemma | Dart |
|  | Jessica | Docherty |
|  | Irina | Ershova |
|  | Alexandra | Firth |
|  | Jeanifer | Gachev |
|  | Leanne | Galloway-Browne |
|  | Jillian | Hanson |
|  | Amelie | Harle |
|  | Farzana | Haque |
|  | Janine | Heeley |
|  | Jane | Hook |
|  | Jane | Hughes |
|  | Alison | Judge |
|  | Emma | Livesey |
|  | Emma | Lundy |
|  | Michael | O'Brian |
|  | Mike | Osborne |
|  | Lauren | Paul |
|  | Mary | Perrin |
|  | Annet | Pillai |
|  | Charlotte | Richardson |
|  | Suzanne | Rogerson |
|  | Olorunda | Rotimi |
|  | Emily | Rudolph |
|  | Jenny | Seligmann |
|  | Hannah | Shanks |
|  | Daniel | Swinson |
|  | Danny | Ulahannan |
|  | Mariah | Vorajee |
|  | Sarah | Wetherop |
|  | Hannah | Wigginton |
|  | Christopher | Williams |
|  | Jawairiya | Zubair |
| **Velindre Hospital** | Rob | Jones (PI) |
|  | Richard | Adams (PI) |
|  | Seema | Arif |
|  | Rebecca | Bethell |
|  | Kathy | Bishop |
|  | Alison | Brewster |
|  | Michael | Brown |
|  | Julie | Broughton |
|  | Lucy | Chestney |
|  | Nikki | Coates |
|  | Sonali | Dasgupta |
|  | Karen | Davies |
|  | Clare | Donnithorne |
|  | Beverley | Eley |
|  | Maria | Evans |
|  | Julie | Graham |
|  | Jill | Halpin |
|  | Robert | Henley |
|  | Lynda | Holman |
|  | Alys | Irving |
|  | Amanda | Jackson |
|  | Emma | John |
|  | Rhianydd | Jones |
|  | Colette | Kemp |
|  | Satish | Kumar |
|  | Claire | Lang |
|  | Cindy | Langford |
|  | Debbie | O'Connor |
|  | Diana | Osman |
|  | Catherine | Matthews |
|  | Ross | McLeish |
|  | James | Morgan |
|  | Phillip | Morgan |
|  | Renata | Poole |
|  | Karen | Pow |
|  | Joanne | Preece |
|  | Cathy | Richards |
|  | Jayne | Richards |
|  | Rosie | Roberts |
|  | Sharon | Rogers |
|  | Jodie | Sherburn |
|  | Lisa | Stafford |
|  | Catherine | Sullivan |
|  | Hana | Thomas |
|  | Anshu | Wadhawan |
|  | Catherine | Watkins |
|  | Hilary | Williams |
|  | Rachel | Williams |
|  | Kay | Wilson |
|  | Rebecca | Wilson |
|  | Cheryl | Worsey |
|  | Charlotte | Young |
| **Christie Hospital** | Michael | Braun (PI) |
|  | Jo | Allen |
|  | Hannah | Arhinful |
|  | Janet | Beech |
|  | Martin | Birch |
|  | Anna | Bowron |
|  | Suzanne | Carter |
|  | Jackie | Connell |
|  | Camille | Cooney |
|  | Rebecca | Cox |
|  | Olive | Craven |
|  | Mark | Cutting |
|  | Hannah | Downs |
|  | Karen | Forshaw |
|  | Chloe | Gawlik |
|  | Jurjees | Hasan |
|  | Sarah | Hughes |
|  | Paul | Husbands |
|  | Mary | Jepson |
|  | Konstantinos-Vellios | Kamposioras |
|  | Rhea | Langeveld |
|  | Catherine | McBain |
|  | Danielle | McCool |
|  | Saifee | Mullamitha |
|  | Monica | Narasimham |
|  | Alkesh | Patel |
|  | Jane | Rogan |
|  | Mark | Saunders |
|  | Sue | Seifi |
|  | Lilly | Simpson |
|  | Greg | Wilson |
|  | Marie | Woolley |
| **Weston Park Hospital** | Joanne | Hornbuckle (PI) |
|  | Jess | Aldred |
|  | Cyper | Allan |
|  | Richard | Allen |
|  | Lynne | Ashmore |
|  | Darrell | Barrott |
|  | Alex | Bradshaw |
|  | Richard | Brown |
|  | Sarah | Brown |
|  | Kevin | Chan |
|  | Rachael | Clarke |
|  | Su | Clark |
|  | Susan | Clenton |
|  | Richard | Crossley |
|  | Alice | Dewdney |
|  | Julia | Disney |
|  | Lynne | Dixon |
|  | Paul | Dodd |
|  | Sarah | Gill |
|  | Elizabeth | Hodgkinson |
|  | Helen | Joyce |
|  | Mandip | Khaira |
|  | Panagiota | Kitsanta |
|  | Jasima | Latif |
|  | Laura | Lee |
|  | Cathryn | Leng |
|  | Tony | Matthew |
|  | Eileen | Marsh |
|  | Aimee | Pendlebury-Worrad |
|  | Megan | Perry |
|  | Simon | Pledge |
|  | Muneeb | Qureshi |
|  | Alison | Redfearn |
|  | Helen | Shulver |
|  | Helen | Slater |
|  | Laura | Smith |
|  | Svetlana | Solovieva |
|  | Catherine | Spalton |
|  | Elisavet | Theodoulou |
|  | Lucy | Walkington |
|  | Christopher | Walls |
|  | Mary | Ward |
|  | Katherine | Williams |
|  | Vanessa | Wilshaw |
|  | Robin | Young |
| **Bristol Haematology & Oncology Centre** | Stephen | Falk (PI) |
|  | Elizabeth | Allison |
|  | Paul | Armenise |
|  | Lindsay | Ball |
|  | Helen | Brooks |
|  | Paula | Byrne |
|  | Carlota | Clemente |
|  | Marc | Coe |
|  | Kiril | Delchev |
|  | Kay | Drury |
|  | Emily | Foulstone |
|  | Elysia | Gower |
|  | John | Helbrow |
|  | Jon | Heywood |
|  | Robert | Hollister |
|  | Kirsten | Hopkins |
|  | Hollie | Jones |
|  | Lauren | Joyce |
|  | Helen | Kingston |
|  | Jayne | Leonard |
|  | Amelia | Lowe |
|  | Catherine | McDonald |
|  | Helen | Monnington |
|  | Katarina | Milutinovic |
|  | Jessica | Nuttall |
|  | Yasmin | Odding |
|  | Ronak | Patel |
|  | Ian | Penwarden |
|  | Peter | Robertson |
|  | Tim | Robinson |
|  | Sharon | Short |
|  | Thomas | Strawson-Smith |
|  | Kirsty | Stevenson |
|  | Hannah | Taylor |
|  | Sandra | Williams (nee Price) |
|  | Eve | Watson |
|  | Axel | Walther |
|  | Angela | Webb |
|  | Tom | Wilson |
|  | Roland | Wynn-Williams |
| **Royal United Hospital** | Emma | De Winton (PI) |
|  | Louise | Medley (PI) |
|  | Tania | Allen |
|  | Rowan | Appleby |
|  | Claire | Barron |
|  | Leigh | Biddlestone |
|  | Hannah | Blades |
|  | Ruth | Brydon-Hill |
|  | Shaolin | Chidavaenzi |
|  | Ashley | Cox |
|  | Christine | Cox |
|  | Claire | Craige |
|  | Jane | Crozier |
|  | Samantha | Curtis |
|  | Michael | Daly |
|  | Claire | Dyke |
|  | Rachael | Exley |
|  | Ioana | Fodor |
|  | Yuko | Francis |
|  | Sharath | Gangadhara |
|  | Amy | Gaunt |
|  | Jenny | Gilbert |
|  | Beatrice | Hamilton |
|  | Leonie | Harrison |
|  | Carly | Laxon-Takooree |
|  | Jill | MacDonald-Burn |
|  | Katarzyna | Machura |
|  | Margaret | Macmillan |
|  | Carey | Milsom |
|  | Kate | Moloney |
|  | Sarah | Murdoch |
|  | Joseph | Needham |
|  | Abigail | Pocock |
|  | Vicki | Portingale |
|  | Bryony | Robertson |
|  | Annie | Taylor |
|  | Eve | Tomlinson |
|  | Tom | Tylee |
|  | Rebecca | Wassall |
|  | Samantha | Williams |
|  | Jess | White |
| **Castle Hill Hospital** | Rajarshi | Roy (PI) |
|  | Abigail | Alford |
|  | Katie | Broadbent |
|  | Amandeep | Dhadda |
|  | Ceri | Fuller |
|  | Laura | Hart |
|  | Lyn | Harrison |
|  | Joanne | Hinchcliffe |
|  | Linda | Hoggarth |
|  | Rhian | Horne |
|  | Dawn | Jones |
|  | Louise | Karsera |
|  | Magdalena | Kolodzieg |
|  | Daniel | Lee |
|  | Lisa | Nix |
|  | Paula | O'Reilly |
|  | Kristian | Plowman |
|  | Karen | Shepherd |
|  | Karen | Stubbs |
|  | Gabrielle | Taft |
|  | Reenee | Tiam |
|  | Adam | Wolstencroft |
| **Victoria Hospital (Blackpool)** | Sin | Lau (PI) |
|  | Charlotte | Armer |
|  | Denise | Bennett |
|  | Caroline | Boardman |
|  | Oliver | Brennan |
|  | Laura | Collins |
|  | Falalu | Danwata |
|  | Alexander Paul | Davies |
|  | Emma | Davies |
|  | Leanne | Davies |
|  | Stacey | Donaldson |
|  | Hannah | Ferguson |
|  | Amanda | Finch |
|  | Julie | Frudd |
|  | Karen | Gratrix |
|  | James | Haston |
|  | Sue | Hesketh |
|  | Sue | Lancaster |
|  | Chris | Pemberton |
|  | Karen | Pollard |
|  | Andrew Jonathan | Potter |
|  | Toni | Purcer |
|  | Ella | Riedel |
|  | Nicola | Slawson |
|  | Shabbir | Susnerwala |
|  | Lauren | Thornborough |
|  | Conor | Wilkinson |
|  | Mark | Wrigley |
| **Royal Preston Hospital** | Sin | Lau (PI) |
|  | Amanda | Alty |
|  | Philippa | Antrobus |
|  | Katherine | Ashton |
|  | David | Barber |
|  | Stephanie | Cornthwaite |
|  | William | Croxford |
|  | Parth | Desai |
|  | Kay | Ellel |
|  | Cassandra | Gleeson |
|  | Louise | Hough |
|  | Krishna | Panchal |
|  | Shakeelah | Patel |
|  | Mark | Pitt |
|  | Sarah | Preston |
|  | Andrew | Martyniak |
|  | Aasif | Motala |
|  | Deborah | Williamson |
|  | Claire | Searle |
|  | Shabbir | Susnerwala |
|  | Dorothy | Walmsley |
|  | Saif | Yousif |
| **Musgrove Park Hospital** | Gihan | Ratnayake (PI) |
|  | Clare | Barlow (PI) |
|  | Jan | Ashcroft |
|  | Hilary | Barlow |
|  | Nita | Beacham |
|  | Erica | Beaumont |
|  | Hannah | Berry |
|  | Becky | Brown |
|  | Clair | Brunner |
|  | Richard | Burgess |
|  | Alison | Chedham |
|  | Hayley | Cornall |
|  | Nicola | Cutmore |
|  | Flora | Darch |
|  | Natasha | Eveleigh |
|  | John | Geraghty |
|  | Fiona | Goodchild |
|  | Emma | Gray |
|  | Clair | Hinton |
|  | Lucy | Howell-Drewett |
|  | Joan | Kemp |
|  | Catherine | Lane |
|  | Fen | Lewen |
|  | Dee | Lewis |
|  | Angela | Locke |
|  | Sue | Mahoney |
|  | Samantha | Northover |
|  | Joanne | Rogers |
|  | Guillermo | Reina-Ruiz |
|  | Joy | Rowe |
|  | Alison | Snell |
|  | Luke | Stephens |
|  | Joanne | Taylor |
|  | Rebecca | Twemlow |
|  | Rebecca | Wallbutton |
|  | Jasmine | Youens |
|  | Robert | Zorica |
|  | Maria | Zietz |
| **Hammersmith Hospital** | Harpreet | Wasan (PI) |
|  | Ilyas | Ali |
|  | Nawa | Amin |
|  | Gareth | Barker |
|  | Michelle | Chen |
|  | Sarah | Chilcott-Burns |
|  | James | Clark |
|  | Susan | Cleator |
|  | Christopher | Coyle |
|  | Andrea | Davis-Cook |
|  | Keyury | Desai |
|  | Matthew | Flook |
|  | Victoria | Harding |
|  | Gillian | Hornzee |
|  | Victoria | Latham |
|  | Luzviminda | Llemit Ramos |
|  | Charles | Lowdell |
|  | Maria | Martinez |
|  | Daniel | Meredith |
|  | Laura | Morland |
|  | Annette | Musallam |
|  | Chynna | Pascual |
|  | Emily | Pickford |
|  | David | Pinato |
|  | Keira | Pudge |
|  | Ramya | Ramaswami |
|  | Azeem | Saleem |
|  | Amalia | Saucan |
|  | Sarah | Stimpson |
|  | Regina | Storch |
|  | Caroline | Ward |
|  | Adrian | Zebrowski |
| **Huddersfield Royal Infirmary** | Jo | Dent (PI) |
|  | Zenab | Ahmed |
|  | Mohammad Irfan | Alam |
|  | Nick | Brown |
|  | Sam | Dale |
|  | Nicky | Daker |
|  | Denise | Hancock |
|  | James | Harris |
|  | Lisa | Horner |
|  | Ibrar | Hussain |
|  | Jeremy | Hyde |
|  | Paula | Gomes |
|  | Rebecca | Jenkins |
|  | Christopher | Knight |
|  | Adam | Mawer |
|  | Mandy | Madigan |
|  | Belinda | McLean |
|  | Sabiha | Ravat |
|  | Hannah | Riley |
|  | Jodie | Rowan |
|  | Simone Deborah | Ryan |
|  | Lisa | Shaw |
|  | Selina | Shaw |
|  | Kathryn | Smith |
|  | Christine | Turner |
|  | Georgina | Turner |
|  | Hayley | Webster |
|  | Tracy | Wood |
| **Northampton General Hospital** | Roshan | Agarwal (PI) |
|  | Sabri | Ahmed |
|  | Caroline | Duncombe |
|  | Tasnim | Ebrahimjee |
|  | Rachel | Gabitass |
|  | Ethelwolda | Goyena |
|  | Andrea | Hillyer |
|  | Jane | Hosea |
|  | Mohammad | Hussain |
|  | Kashif | Jarral |
|  | Andrea | Jones |
|  | Andrea | Kempa |
|  | Adnan | Masood |
|  | Craig | Macmillan |
|  | James | Maloy |
|  | Katherine | McGrath |
|  | Jan | Miles |
|  | Onyinye | Ndefo |
|  | Paula | O'Connell |
|  | Malgorzata | Polnik |
|  | Ehsan | Rahman |
|  | Shahriar Mohammed | Reza |
|  | Sharon | Ryan |
|  | Simon | Stapley |
|  | Elizabeth | Tee |
|  | Lenka | Zvirinska |
| **Pinderfields Hospital** | Iva | Damyanova (PI) |
|  | Ashraf | Alkhaldi (PI) |
|  | Gireesh | Kumaran (PI) |
|  | Usman | Ahmad |
|  | Aneeka Shubnum | Altaf |
|  | Julie | Ball |
|  | Louise | Benton |
|  | Kevin | Birbeck |
|  | Lynsey | Bourner |
|  | Richard | Bowers |
|  | Hollie | Brooke |
|  | Ellis | Burton |
|  | Julie | Burton |
|  | Deborah | Cooper |
|  | Elizabeth | Clayton |
|  | Jane | Eastwood |
|  | Aimee | Fletcher |
|  | Rebecca | Foster |
|  | Darren | Gomersall |
|  | Hassan | Hameed |
|  | Aimee | Hayton-Bott |
|  | Charlotte | Hirst |
|  | Claire | Hutsby |
|  | Andrew M | Jackson |
|  | Annette | Jones |
|  | Konstantinos-Vellios | Kamposioras |
|  | Patricia | Kane |
|  | Tracey | Lowry |
|  | Stephanie | Lupton |
|  | Joanna | Lyle |
|  | Kate | Norton |
|  | Ganesh | Radhakrishna |
|  | Vishal | Ramdhani |
|  | Muhammad Bilal | Razzaq |
|  | Ayesha | Sheikh |
|  | Hira | Yousif |
| **Beatson West of Scotland Cancer Centre** | Janet | Graham (PI) |
|  | Tareq | Abdullah |
|  | Ghada | Al-Salih |
|  | Martin | Ball |
|  | Karen | Bell |
|  | Anette | Charlick |
|  | Maureen | Connolly |
|  | Jill | Dempster |
|  | Alan | Foulis |
|  | Paula | Henry-Stephenson |
|  | Jill | Graham |
|  | Lesley | Hickey |
|  | Sandra | Jenkins |
|  | Sai Juan | Jia |
|  | Jennifer | Keith |
|  | Donna | Kelly |
|  | Audrey | Leonard |
|  | Gail | Lynch |
|  | Alex | McDonald |
|  | Jordan | McGill |
|  | Anne | McKillop |
|  | Austin | McInnes |
|  | Fiona | McQueen |
|  | Nazia | Mohammed |
|  | Paul | Mooney |
|  | Maria | Nygren |
|  | Shilpa | Thapar |
|  | Kirsty | Ross |
|  | Patricia | Roxburgh |
|  | Pavlina | Spiliopoulou |
|  | Eileen | Soulis |
|  | Kirsteen | Stuart |
|  | Rasheed | Syed |
|  | Ashita | Waterston |
|  | Cheryl | Wilson |
| **Ysbyty Gwynedd** | Catherine | Bale (PI) |
|  | Kelly | Andrews |
|  | Naomi | Boyle |
|  | Claire | Fuller |
|  | John | Grant |
|  | Emma | Hall |
|  | Anna | Mullard |
|  | Wendy | Saxton |
|  | Nick | Stuart |
|  | Alice | Thomas |
|  | Linzi | Williams |
|  | Rachel | Williams |
| **Withybush General Hospital** | Sarah | Gwynne (PI) |
|  | Maung | Moe (PI) |
|  | Fawwaz | Arikat |
|  | Denisa | Asandei |
|  | Sandra | Evans |
|  | Eirianydd | Garrard |
|  | Sophie | Glynn-Williams |
|  | Colette | Griffiths |
|  | Rachel | Hughes |
|  | Catherine | MacPhee |
|  | John | Murphy |
|  | Kirsty | Pope |
|  | Rocio | Riba |
|  | Sally-Ann | Rolls |
|  | Abigail | Taylor |
|  | Carol | Thomas |
|  | Helen | Thomas |
|  | Vallipuram | Vigneswaran |
| **Aberdeen Royal Infirmary** | Leslie | Samuel (PI) |
|  | Fay | Annison |
|  | Sharon | Armstrong |
|  | Abimbola | Barango |
|  | Balazs | Binnyei |
|  | Gillian | Brand |
|  | Kay | Campbell |
|  | Angie | Cheyne |
|  | Michael | Christie |
|  | Kathryn | Connolly |
|  | Pat | Cooper |
|  | Amber | Johnson |
|  | Susan | Martin |
|  | Celia | Meneses |
|  | Graeme | Murray |
|  | Nicola | Price |
|  | Sue | Rodwell |
|  | Mhairi | Scott |
|  | Margaret | Smith |
|  | Bartosz | Was |
|  | Mehmood | Zaidi |
|  | Ishtiaq | Zubairi |
| **Cheltenham General Hospital** | Kim | Benstead (PI) |
|  | Jaqueline | Aberdeen |
|  | Rehana | Bakawala |
|  | Sarah | Beazer |
|  | Colin | Binks |
|  | Lucy | Blake |
|  | Bethan | Cartwright |
|  | Samuel | Croly |
|  | Lin | Crossley |
|  | Rachel | Durrant |
|  | David | Farrugia |
|  | Janet | Forkes |
|  | Emma | Gilbert |
|  | Fabrizio | Mauri |
|  | Elaine | Pratten |
|  | Elisabeth | Read |
|  | Nick | Reed |
|  | Rachel | Sayers |
|  | Neil | Shepherd |
|  | Stephen | Shepherd |
|  | Jennifer | Smith |
|  | Sarah | Stanley |
|  | Catherine | Stuart-Grumbar |
|  | Bilal | Topia |
|  | Kate | Trigg-Hogarth |
| **Clatterbridge Centre for Oncology** | Nasim | Ali (PI) |
|  | Wesley | Artist |
|  | Shaker | Abdallah |
|  | Alexandra | Bailey |
|  | Danielle | Campbell |
|  | Maggie | Cantrell |
|  | Joanne | Cliff (nee Mooney) |
|  | Thomas | Davies |
|  | Helen | Flint |
|  | Amy | Ford |
|  | Barbara | King |
|  | Ayman | Madi |
|  | Samah | Massalha |
|  | Laura | McAllister |
|  | Amir | Montazeri |
|  | Joanne | Mullen |
|  | Julie | O'Hagan |
|  | Anna | Olsson-Brown |
|  | Katharine | Pelton |
|  | Kelly | Richardson |
|  | Sandra | Robinson |
|  | Joseph | Sacco |
|  | Sarah | Stuart |
|  | Hollie | Wilson |
|  | Pembe | Yesildag |
|  | Mariah | Zavery |
| **Royal Devon and Exeter Hospital** | Melanie | Osborne (PI) |
|  | Kizzy | Baines |
|  | Tamika | Chapter |
|  | Elizabeth | Davey |
|  | Susan | Downer |
|  | Dawn | Edwards |
|  | Theresa | Lawless |
|  | James | Leavy |
|  | Mark | Napier |
|  | Emma | Robjohns |
|  | Patrick | Sarsfield |
|  | Ingrid | Seath |
|  | Shirley | Todd |
|  | Jane | Thompson |
|  | Fiona | Walters (nee Hall) |
|  | Claire | Webb |
|  | Julia | Weston |
| **Southampton General Hospital** | Tim | Iveson (PI) |
|  | Liane | Armstrong |
|  | Andrew | Bateman |
|  | Adrian | Bateman |
|  | Emma | Brown |
|  | Holly | Burton |
|  | Tracey | Callen |
|  | Bethany | Caruana |
|  | Caroline | Chau |
|  | Tracey | Day |
|  | Efe | Evbuomwan |
|  | Meg | Gale |
|  | Julie | Gwilt |
|  | Sara | Hosseini-Moein |
|  | Alice | Johnson |
|  | Leah | Long |
|  | Steve | McKenzie |
|  | Charlotte | Rees |
|  | Rasha | Said |
| **University College Hospital** | John | Bridgewater (PI) |
|  | Adrienne | Abioye |
|  | Mahfuja | Ahmed |
|  | Shamima | Akther |
|  | Maise | Al Bakir |
|  | Adelaide | Austin |
|  | Holly | Baker |
|  | Jaytee | Barnett |
|  | Nina | Bason |
|  | Isabelle | Brown |
|  | Alexa | Childs |
|  | Louise | Coyle |
|  | Patricia | Danaswamy |
|  | Kanishka | Dissansayke |
|  | Rosina | Donovan |
|  | Lola | Enemuwe |
|  | Victor | Eneh |
|  | Gabrielle | Gould |
|  | Todd | Gumbleton |
|  | Selina | Gurung |
|  | Gemma | Hector |
|  | Sonya | Hessey |
|  | Daniel | Hochhauser |
|  | Sabrina | Holohan |
|  | Michelle | Hung |
|  | Georgios | Imseeh |
|  | Adoracion | Jayme |
|  | Sarah | Kerr |
|  | Khurum | Khan |
|  | Jennifer | Laude |
|  | Xiao | Lu |
|  | Gina | Margai |
|  | Katie | Matthews |
|  | Eman | Mohamad |
|  | Fatima | Mohamed |
|  | Sam | Morris |
|  | Anna | Nikopoulou |
|  | Mayur | Patel |
|  | Maria | Power |
|  | Prakash | Rao |
|  | Manuel | Rodriguez-Justo |
|  | Derya | Sahin |
|  | Kai Keen | Shiu |
|  | Luke Owen | Steventon |
|  | Mark | Sunga |
|  | Hinesh | Tailor |
|  | Anisa | Tariq |
|  | Varji | Thayalan |
|  | Jennifer | Thomas |
|  | Christopher | Wanstall |
|  | Kristian | Warnes |
|  | Christopher | Whitton |
|  | Georgina | Wood |
| **Monklands Hospital** | Lisa | Rogers (PI) |
|  | Anne | McKillop (PI) |
|  | Ashita | Waterston (PI) |
|  | Paula | Botham |
|  | June | Carr |
|  | Louise | Devlin |
|  | Katie | Douglas |
|  | Grainne | Dunn |
|  | Mohammed | El-Abdullah |
|  | Lynn | Glass |
|  | Kirsteen | Hamill |
|  | Susan | Hastings |
|  | Rebecca | Heron |
|  | Chloe | MacDonald |
|  | Steven | Marshall |
|  | Laura | Miller |
|  | Geradline | O'Dowd |
|  | Aqilah | Othman |
|  | Diana | Park |
|  | Angela | Scullion |
|  | Denise | Vigni |
|  | Kai | Yahya |
| **Charing Cross Hospital** | Harpreet | Wasan (PI) |
|  | Thalia | Afxentiou |
|  | Riz | Ahmed |
|  | Melloney | Allnutt |
|  | Gareth | Barker |
|  | Abigail | Caldow |
|  | Jolene | Carioni |
|  | Sarah | Chilcott-Burns |
|  | Andrea | Davis-Cook |
|  | Yomi | Fatola |
|  | Chee | Goh |
|  | Dorothy | Gujral |
|  | Gillian | Hornzee |
|  | Eleni | Josephides |
|  | Charlotte | Kelly |
|  | Daleep | Kumar |
|  | Priya | Limbu |
|  | Luzviminda | Llemit Ramos |
|  | Charles | Lowdell |
|  | Sophia | Magwaro |
|  | Rochelle | McIntyre |
|  | Philippa | Nutkins |
|  | Shola | Ogegbo |
|  | Anna | Osei-Kofi |
|  | Susan | Ramsey |
|  | Pippa | Riddle |
|  | Amalia | Saucan |
|  | Helen | Saxby |
|  | Chantelle | Simpson |
|  | Aspa | Spyrou |
|  | Kirsty | Tunna |
|  | Iman | Yahya |
|  | Adrian | Zebrowski |
| **Churchill Hospital, Oxford** | Tim | Maughan (PI) |
|  | David | Badcock |
|  | Magdalena | Benysek |
|  | Rosita | Broderick |
|  | Anne | Butterfield |
|  | Evelyn | Chan |
|  | Philip | Charlton |
|  | David | Church |
|  | Richard | Cousins |
|  | Louise | Cowen |
|  | Joanne | Davies |
|  | Steven | Davis |
|  | Alfonso | Gonzalez Blas |
|  | Will | Goodman |
|  | Nikki | Hayward |
|  | Clare | Jacobs |
|  | Patrycja | Jastrzebska |
|  | Evanthia | Komninidou |
|  | Jonathan | Lau |
|  | Carolina | Lepiato |
|  | Clare | Marken |
|  | Kerrie | Marston |
|  | Mark | Middleton |
|  | Ann | Murphy |
|  | Rebecca | Muirhead |
|  | Adrian | Nicholson |
|  | Robin | Peach-Toon |
|  | Navin | Pol |
|  | Sally | Rich |
|  | Nicola | Stoner |
|  | James | Wakelin |
|  | Lai Mun | Wang |
|  | Andrew | Weaver |
|  | Sandie | Wellman |
|  | Anthony | Wilson |
|  | Rebecca | Wiltshire |
|  | Martha | Woodward |
|  | Kirsten | Wynn |
| **Leicester Royal Infirmary** | Anne | Thomas (PI) |
|  | Will | Steward (PI) |
|  | Elizabeth | Andrzejewski |
|  | Tracey | Alexander |
|  | Sarah | Attridge |
|  | Julie | Barlow |
|  | Theresa | Beaver |
|  | Amy | Branson |
|  | Meera | Chauhan |
|  | Aurora | Del Pozo |
|  | Hadia | Haque |
|  | Hannah | Holdsworth |
|  | Rahima | Ibrahim |
|  | Chinenye | Iwuji |
|  | Mohammed | Karolia |
|  | Lydianne | Lock |
|  | Mohammed | Mahgoub |
|  | Adrian | Nicholson |
|  | Ahmed | Osman |
|  | Katherine | Perkins |
|  | Sarah | Porter |
|  | Thiaghrajon | Sridhar |
|  | Judith | Underwood |
|  | Balaji | Varadhan |
|  | Julia | Walker |
|  | Kevin | West |
|  | Joanna | Wood |
| **Raigmore Hospital** | Walter | Mmeka (PI) |
|  | Anglise | Addison |
|  | Seonaid | Arnott |
|  | Karen | Callum |
|  | Denise | Campbell |
|  | Fiona | Campbell |
|  | Kay | Kelly |
|  | Alison | Macdonald |
|  | Angela | Macgregor |
|  | Carol | Macgregor |
|  | Zoe | Maciver |
|  | Laura | Maclennan |
|  | Jude | Madeleine |
|  | Melanie | McIlroy |
|  | Mary | McKenzie |
|  | Neil | McPhail |
|  | Alison | Nicholls |
|  | Marion | Paterson |
|  | Leslie | Samuel |
|  | Georgina | Simpson |
|  | Glenda | Sinclair |
|  | Feng Yi | Soh |
|  | Grant | Stenhouse |
|  | Joan | Stewart |
|  | Una | Taylor |
|  | Zoe | Urquhart |
| **Victoria Hospital (Kirkcaldy)** | Sally | Clive (PI) |
|  | Brian | Adamson |
|  | Julie | Aitken |
|  | John | Brush |
|  | Rebecca | Cain |
|  | Lesley | Cargill |
|  | Shona | Cheyne |
|  | Clare | Cliff |
|  | Hazel | Cree |
|  | Karen | Gray |
|  | Sophie | Iwanikiw |
|  | Fiona | Johnston |
|  | Alastair | Matthews |
|  | Wendy | McCorry |
|  | Catriona | Mclean |
|  | Fiona | Murdoch |
|  | Ibrahim | Nawroz |
|  | Julie | Penman |
|  | Anna | Scott |
|  | Maria | Simpson |
|  | Deepak | Subedi |
|  | Jennifer | Tait |
|  | Michelle | Tingley |
|  | Linzi | Wilson |
| **Princess Alexandra Hospital (Harlow)** | John | Bridgewater (PI) |
|  | Gemma | Cook |
|  | Amelia | Daniel |
|  | Venkatesh | Gajapathy |
|  | Evelyn | Holmes |
|  | Tayo | Jaiyesimi |
|  | Joanne | Kellaway |
|  | Teresa | Light |
|  | Lucinda | Melcher |
|  | Cait | Rees |
|  | Vasi | Sundaresan |
| **Royal Surrey County Hospital** | Tony | Dhillon (PI) |
|  | Mazhar | Ajaz |
|  | Nawa | Amin |
|  | Humyraa | Aziz |
|  | Izhar | Bagwan |
|  | Catherine | Blake |
|  | Fiona | Butler |
|  | Penny | Champion |
|  | Karen | Chan |
|  | Sebastian | Cummins |
|  | Tineke | Edmunds |
|  | Sharadah | Essapen |
|  | Andrew | Furness |
|  | Laura | Gordon |
|  | Di | Grainger |
|  | Helen | Graves |
|  | Imogen | Heenan |
|  | Kirsty | Horwood |
|  | Daniel | Jennings |
|  | Natasha | Kamboh |
|  | Aga | Kehinde |
|  | Karla | Lee |
|  | Sibylle | Lintott |
|  | Gaybrielle | Livingstone |
|  | Cheryl | Marriott |
|  | Catherine | Medcalf |
|  | Aruna | Medisetti |
|  | Mahomed | Moosa |
|  | Gayathri | Nagarajan |
|  | Sarah | Oakes |
|  | Sue | Sargent |
|  | Alexandra | Stewart |
|  | Hasina | Thandar |
|  | Claire | Thompson |
|  | Katharine | Webb |
|  | Rosalyne | Westley |
|  | Julia | Whittle |
|  | Julie | Wilkinson |
|  | Rebecca | Wills |
| **St Helens Hospital** | Zahed | Khan (PI) |
|  | Rachel | Cassidy |
|  | Jenny | Cotton |
|  | Lisa | Dobson |
|  | Nicola | Hornby |
|  | Sheila | Kelly |
|  | Amanda | McCairn |
|  | Jeanette | Ribton |
|  | Michelle | Robinson |
|  | Carol | Ross |
|  | Victoria | Thomas |
| **Chesterfield Royal Hospital** | Vanessa | Wilshaw (PI) |
|  | Ibrahim | Al-Modaris |
|  | Rebecca | Clark |
|  | Aurora | Del Pozo |
|  | Alice | Dewdney |
|  | Nicky | Ford |
|  | Rachel | Gascoyne |
|  | Neeta | Gogna |
|  | Charlotte | Hoult |
|  | Emma | Hudson |
|  | Kelly | Pritchard |
|  | Martin | Shepherd |
|  | Lesley | Stevenson |
|  | Danesh | Taraporewalla |
|  | Julie | Toms |
|  | Katie | Wallace |
|  | Julie | Whitehead |
|  | Lucinda | Wilson |
| **Ipswich Hospital** | Gopalakrishnan | Srinivasan (PI) |
|  | Zoltan | Szucs (PI) |
|  | Deborah | Abrams |
|  | Debbie | Austin |
|  | Carlos | Gonzalez |
|  | Matthew | Howlett |
|  | Natalie | Lloyd |
|  | Rita | Ng |
|  | Paul | Ridley |
|  | Kirubah | Selvaraj |
|  | Liz | Sherwin |
|  | Bamini | Sivarajah |
|  | Susan | Upson |
|  | Angharad | Williams |
|  | Jason | Wong |
| **Royal Hampshire County Hospital** | Luke | Nolan (PI) |
|  | Louise | Beattie |
|  | Julie | Conti |
|  | Duncan | Cooke |
|  | Victoria | Corner |
|  | Adrienn | Fazekasne Fulep |
|  | Angela | Frith |
|  | Julie | Gwilt |
|  | Samantha | Hammond |
|  | Liz | Happle |
|  | Lesley | Hollister |
|  | Roger | Hudson |
|  | Abigail | Hughes |
|  | Lauriane | Kerwood |
|  | Matthew | Pitt |
|  | Balvinder | Shoker |
|  | Rao | Vuyyuru |
| **Peterborough City Hospital** | Catherine | Jephcott (PI) |
|  | Terri-Anne | Baker |
|  | Helen | Bowyer |
|  | Kerrie | Cavanagh |
|  | Rebecca | Chilvers |
|  | Marilyna | Chong |
|  | Laura | Costello |
|  | Abigail | Hollingdale |
|  | Steph | Lawrence |
|  | Heather | Maccoll |
|  | Carla | Martino |
|  | Claire | Palombo |
|  | Stuart | Richmond |
|  | Richard | Skells |
|  | Laura | Simon |
|  | Claire | Snowden |
|  | Lisa | Wilde |
|  | Louise | Wilmer |
| **Calderdale Royal Hospital** | Jo | Dent (PI) |
|  | Mohammad Irfan | Alam |
|  | Nick | Brown |
|  | Nicky | Daker |
|  | Sam | Dale |
|  | Denise | Hancock |
|  | James | Harris |
|  | Lisa | Horner |
|  | Jeremy | Hyde |
|  | Rebecca | Jenkins |
|  | Christopher | Knight |
|  | Mandy | Madigan |
|  | Adam | Mawer |
|  | Belinda | McLean |
|  | Sabiha | Ravat |
|  | Hannah | Riley |
|  | Jodie | Rowan |
|  | Simone Deborah | Ryan |
|  | Lisa | Shaw |
|  | Selina | Shaw |
|  | Kathryn | Smith |
|  | Christine | Turner |
|  | Georgina | Turner |
|  | Hayley | Webster |
|  | Tracy | Wood |
| **Derriford Hospital** | David | Sherriff (PI) |
|  | Rebecca | Aaron |
|  | Bridget | Aire |
|  | Baffour | Amo-Takyi |
|  | Erin | Brennan |
|  | Lucy | Cadmore |
|  | Leonie | Eastlake |
|  | Laura | Evenden |
|  | Kay | Facey |
|  | Olivia | Fraser |
|  | Julie | Froud |
|  | Bojidar | Goranov |
|  | Irene | Harvey |
|  | Maggie | Kalita |
|  | Sarah | Kingdon |
|  | Mike | Marner |
|  | Laura | Marks |
|  | Susan | McFarlane |
|  | Chelsea | Morton |
|  | Anna | Mucha |
|  | Sarah | Prance |
|  | Olivia | Reed-Poysden |
|  | Peter | Sankey |
|  | Helen | Smith |
| **Macclesfield District General Hospital** | Victoria | Lavin (PI) |
|  | Ganesh | Radhakrishna (PI) |
|  | Catherine | McBain (PI) |
|  | Victoria | Adinkra |
|  | Dane | Bradwell |
|  | Lisa | Brookes |
|  | Helen | Burns |
|  | Nicola | Dawson |
|  | Catherine | Fenson |
|  | Lisa | Hardstaff |
|  | Abbi | Henderson |
|  | Christy | Henderson |
|  | Pippa | Hill |
|  | Debra | Jowle |
|  | Mark | Lawrence |
|  | Joanna | Longden |
|  | Nicola | Lunt |
|  | Marilyn | McCurrie |
|  | Karen | Rotchell |
|  | Barbara | Townley |
|  | Helen | Wassall |
|  | Julie | Whitehead |
|  | Lesley | Wilkinson |
|  | Iain | Woodhouse |
| **Torbay District General Hospital** | Nangi | Lo (PI) |
|  | Michele | Allison |
|  | Kenneth | Almedilla |
|  | Emmie | Arbury |
|  | Lauren | Blunt |
|  | Jo | Blurton |
|  | Catherine | Brookman |
|  | Ian | Buley |
|  | Shelley | Chamberlain |
|  | Stacey | Davies |
|  | Angela | Foulds |
|  | Meadow | Fisher-Crisp |
|  | Joanne | Garfield-Smith |
|  | Petra | Gee |
|  | Caera | Good |
|  | Hannah | Griffin |
|  | Andrew | Harford-Brown |
|  | Prithvi | Jampana |
|  | Ingrid | Koehler |
|  | Tyler | Lowe |
|  | Sally | Maddison |
|  | Mitchell | McMillan |
|  | Louise | Medley |
|  | Lyn | Micklewright |
|  | Louise | Paatz |
|  | Maeve | Pomeroy |
|  | Helen | Randall |
|  | Fleur | Rogers |
|  | Lorraine | Thornton |
|  | Christine | Tsang |
|  | Elaine | Vandecandalaere |
|  | Sarah | Wright |
| **Addenbrooke's Hospital** | Hugo | Ford (PI) |
|  | Athar | Ahmad |
|  | Alexandra | Azevedo |
|  | Lesley | Bennett |
|  | Elizabeth | Blake |
|  | Mark | Bolton |
|  | Rebecca | Bradley |
|  | Jane | Bushen |
|  | Joanna | Calder |
|  | Anita | Chhabra |
|  | Kathy | Chin |
|  | Sarah | Clark |
|  | Joseph | Gallagher |
|  | Svitlana | Iyevkova |
|  | Rashmi | Jadon |
|  | Catherine | Jephcott |
|  | Natalie | Jones |
|  | Hannah | Loveday |
|  | Jane | Macdonald |
|  | Betania | Mahler-Araujo |
|  | Debra | Mansergh |
|  | Ultan | McDermott |
|  | Lindsay | Piper |
|  | Amy | Strong |
|  | Catherine | Thorbinson |
|  | Saji | Victor |
|  | Naval | Vyse |
|  | Amanda | Walker |
|  | Emma | Wong |
|  | Zsuzsa | Zaborszky |
| **Guy's Hospital (London)** | Paul | Ross (PI) |
|  | Samantha | Barrett |
|  | Eva | Batovska |
|  | Jessica | Brady |
|  | Maribel | Boyce |
|  | Laura | Camburn |
|  | Lorna | Caplis |
|  | Noan Minh | Chall |
|  | Jason | Chow |
|  | Chi Yee | Chung |
|  | Sophie | Clark |
|  | Sarah | Cleary |
|  | Victoria | Donovan |
|  | Sandra | Esteban Moreno |
|  | Adrienn | Fazekasne Fulep |
|  | Lucy | Featherstone |
|  | Michael | Flanagan |
|  | Laura | Green |
|  | Sara | Hulf |
|  | Arun | Karnad |
|  | Sara | Kazemzadeh |
|  | Vevangaune | Ketjiperue |
|  | Choi Chin | Lau |
|  | Nick | Maisey |
|  | Simranjit | Mehta |
|  | Ngozi | Muoneke |
|  | Theodorah | Nago |
|  | Rita | Njoku |
|  | Vitalis | Nwokorie |
|  | Temi | Olusi |
|  | Kishen | Patel |
|  | Amy | Quinn |
|  | Catherine | Rogers |
|  | Hannah | Rush |
|  | Susie | Slater |
|  | Anita | Soma |
|  | Chara | Stavraka |
|  | Harriet | Waine |
|  | Sally | Walker |
| **St Georges Hospital (London)** | Fiona | Lofts (PI) |
|  | Doraid | Alrifa |
|  | Nia | Alsamarrai |
|  | Jason | Chow |
|  | Alice | Dainty |
|  | Lorette | Ffolkes |
|  | Caroline | Finlayson |
|  | Claire | Gilmartin |
|  | Anne | Haldeos |
|  | Sam | Hollingworth |
|  | Geoffrey | Howell |
|  | Robert | Ingham |
|  | Kay | Laurent |
|  | Vitalis | Nwokorie |
|  | Antonio | Pesino |
|  | Mark | Quarrell |
|  | Agne | Sekmokaite |
|  | Jesusa | Toledo |
| **Wrexham Maelor Hospital** | Simon | Gollins (PI) |
|  | Stacy | Ackerley |
|  | Ashraf | Alkhaldi |
|  | Kelly | Andrews |
|  | Rachel | Davies |
|  | Alistair | Ellis-Jones |
|  | Emma | Hall |
|  | Rachel | Hughes |
|  | Ravi | Kodavatiganti |
|  | Arwel | Lloyd |
|  | Bethan Wyn | Owen |
|  | Beryl | Roberts |
|  | Charley-Anne | Rutter |
|  | Jane | Stockport |
|  | Gemma | Szabo |
|  | Ian | Walker |
|  | Claire | Watkins |
|  | Glesni | Williams |
|  | Linzi | Williams |
| **Glan Clwyd Hospital** | Simon | Gollins (PI) |
|  | Elizabeth | Allan |
|  | Jill | Andrews |
|  | Kelly | Andrews |
|  | Lisa | Ashley |
|  | Llinos | Davies |
|  | Rachel | Davies |
|  | Clair | Domeney |
|  | Sarah | Evans |
|  | Emma | Hall |
|  | Jane | Heron |
|  | Ravi | Kodavatiganti |
|  | Joanne | Lewis |
|  | Arwel | Lloyd |
|  | Carey | Macdonald-Smith |
|  | Claire | McGregor |
|  | Bethan Wyn | Owen |
|  | Tracy | Parry-Jones |
|  | Fiona | Redmond |
|  | Beryl | Roberts |
|  | Charley-Anne | Rutter |
|  | Libby | Thackray |
|  | Ian | Walker |
|  | Jill | Westlake-Guy |
|  | Linzi | Williams |
|  | Stephanie | Wynne |
| **James Cook University Hospital** | Nick | Wadd (PI) |
|  | Andrea | Boyce |
|  | Alison | Chilvers |
|  | Anthony | Donnelly |
|  | Helen | Dunn |
|  | Vicky | Hanlon |
|  | Charlotte | Jacobs |
|  | Steven | Liggett |
|  | Craig | Mower |
|  | Lisa | Peacock |
|  | Jacqueline | Richards |
|  | Agnieszka | Skotnicka |
|  | Danielle | Sweeney |
|  | Jane | Thompson |
|  | Hans | Van der Voet |
|  | Gill | Wheater |
|  | David | Wilson |
|  | Jason | Wong |
| **Poole Hospital** | Amelie | Harle (PI) |
|  | Tamas | Hickish (PI) |
|  | Michael | Adrio |
|  | Maria | Alban |
|  | Julian | Alexander |
|  | Lyn | Allen |
|  | Mary | Apps |
|  | Beth | Aubrey |
|  | Helen | Bradley |
|  | Savina | Elitova |
|  | Daniel | Fielding |
|  | Maxine | Flubacher |
|  | Deborah | Forster |
|  | Melanie | Foster |
|  | Louise | Heckford |
|  | Jill | Hobson |
|  | Hannah | James |
|  | Min Yee | Lee |
|  | Helen | Morling |
|  | Victoria | Osborne |
|  | Sharon | Power |
|  | Victoria | True |
|  | Craig | Vincent |
|  | Roger | Wheelwright |
| **Royal Cornwall Hospital** | Richard | Ellis (PI) |
|  | Linda | Allsop |
|  | Nicholas | Ashley |
|  | Kerry | Atkinson |
|  | Nigel | Bailey |
|  | Thea | Barlow |
|  | Kayleigh | Bennett |
|  | Carolyn | Brode |
|  | Thomas | Cornell |
|  | Alexander | Dengler |
|  | Emma | Duley |
|  | Sophia | Eloi |
|  | Caroline | Goddard |
|  | Aaron | Gould |
|  | Anne | Griffiths |
|  | Karina | Harris |
|  | Peter | Helliwell |
|  | Claire | Hill |
|  | Louise | Johns |
|  | Tinnaya | King |
|  | Samantha | Lomax |
|  | Kirsty | Maclean |
|  | John | Madine |
|  | Joe | Mathew |
|  | John | McGrane |
|  | Fiona | Minear |
|  | Sharon | Moore |
|  | Anna | Oakes |
|  | Caroline | Parnell |
|  | Kerena | Partridge |
|  | Sallyanne | Platt |
|  | Kirsty | Prout |
|  | William | Pynsent |
|  | Rebecca | Rogers |
|  | Jenifer | Row |
|  | Laura | Royle |
|  | Johanna | Skewes |
|  | David | Smith |
|  | Darren | Snell |
|  | Luke | Townley |
| **Royal Free Hospital** | Daniel | Krell (PI) |
|  | Astrid | Mayer (PI) |
|  | Tahmin | Ahmed |
|  | Ian | Clark |
|  | Jen | Fraser-Fish |
|  | Roopinder | Gillmore |
|  | Sara | Hamilton |
|  | Ben | Marks |
|  | Leah | Meaden |
|  | Aarti | Nandani |
|  | Tesha | Suddason |
|  | Sharon | Thompson |
|  | Elizabeth | Woodford |
| **South Tyneside District Hospital** | Ashraf | Azzabi (PI) |
|  | Amy | Burns |
|  | Kumud | Jain |
|  | Judith | Moore |
|  | Ruth | Tindle |
| **St Bartholomews Hospital (London)** | David | Propper (PI) |
|  | Waheeda | Abida |
|  | Hayley | Blackgrove |
|  | Joanne | Chin-Aleong |
|  | Nikolaos | Diamantis |
|  | Resmi | Jayachandran |
|  | Sumaiya | Kamora |
|  | Cheryl | Lawrence |
|  | Alia | Mahboob |
|  | Juan | Navarro |
|  | Tanjil | Nawaz |
|  | Pratistha | Panday |
|  | Hannah | Payne |
|  | Stephen | Russell |
|  | Sarah | Slater |
| **Yeovil District Hospital** | Andrew | Allison (PI) |
|  | Erica | Beaumont (PI) |
|  | Matthew | Sephton (PI) |
|  | Joanna | Allison |
|  | Zenaida | Armstrong |
|  | Claire | Barron |
|  | Nigel | Beer |
|  | Kate | Beesley |
|  | Edwin | Cooper |
|  | Sarah | De Bruijn |
|  | David | Donaldson |
|  | Tracey | Duckett |
|  | Adam | Edwards |
|  | Shirley | Fox |
|  | Karen | Flynn |
|  | Michelle | Kotze |
|  | Michaela | Nock |
|  | Jess | Perry |
|  | Lucy | Pippard |
|  | Kerry | Rennie |
|  | Amber | Rowsell |
|  | Rufus | Smith |
|  | Lesley | Thomas |
|  | Barbara | Williams-Yesson |
| **Lincoln County Hospital** | Zuzana | Stokes (PI) |
|  | Antoinette | Adu |
|  | Suzanne | Archer |
|  | Sarah | Bell |
|  | Jayne | Borley |
|  | Sarah | Coombs |
|  | Olesya | Francis |
|  | Annette | Hilldrith |
|  | Kathryn | Hoare |
|  | Carol | Lockwood |
|  | Maryanne | Okubanjo |
|  | Rhiannan | Pegg |
|  | Manuel | Ruiz-Echarri |
|  | Thomas | Sheehan |
|  | Anuradha | Sheth |
|  | Andrew | Sloan |
|  | Caroline | Taylor |
|  | Ruth | Thoy |
|  | Alyson | Wilson |
| **Maidstone Hospital** | Mark | Hill (PI) |
|  | Doraid | Alrifa |
|  | Elizabeth | Angus |
|  | Paulette | Basham |
|  | Lisa | Brown |
|  | Tracey | Chambers |
|  | Alison | Davison |
|  | Jackie | Evans |
|  | Sanjina | Kathuria |
|  | Samantha | Kestenbaum |
|  | Tiana | Kordbacheh |
|  | Satish | Kumar |
|  | Barbara | LeBrocq |
|  | Gemma | McCormick |
|  | Christos | Mikropoulos |
|  | Ian | Pamphlett |
|  | Joanne | Patterson |
|  | Caroline | Rodger |
|  | Holly | Slater |
|  | Charlotte | Stevens |
|  | Jeff | Summers |
|  | Alicia | Synowiec |
|  | Katy | Taylor |
|  | Lisa | Tribe |
| **Nottingham University Hospitals** | Cristina | Lopez Escola (PI) |
|  | Rebecca | Ashton |
|  | Suha | Atabani |
|  | Alex | Blades |
|  | Emma | Blades |
|  | Lauren | Blackburn |
|  | Pauline | Brookes |
|  | Eliot | Chadwick |
|  | Caroline | Coulson |
|  | Michelle | Cunnell |
|  | James | Donworth |
|  | Jade | Eggleton |
|  | Susan | Elliott |
|  | Joanne | Hobbs |
|  | Shaymaa | Hosni |
|  | Laura | Kirk |
|  | Emma | Marshall |
|  | Balwir | Matharoo-Ball |
|  | Kayleigh | Mills |
|  | Jamie | Mills |
|  | Jeanette | Mulhurn |
|  | Karen | Newcombe |
|  | Vanessa | Potter |
|  | Tin | Sang-Tsang |
|  | Rosalind | Roberts |
|  | Maria | Scott |
|  | Rafael | Silverman |
|  | Ananth | Sivanandan |
|  | Tania | Slater |
|  | Anita | Stevenson |
|  | Richard | Swinden |
|  | Jackie | Worville |
|  | Georgina | Walker |
|  | Andrew | Wright |
| **Hinchingbrooke Hospital** | Cheryl | Palmer (PI) |
|  | Shilamba | Bramham |
|  | Sue | Donnelly |
|  | Simon | Duke |
|  | Vanessa | Goss |
|  | Beverley | Haynes |
|  | Rebecca | Lam |
|  | Elizabeth | Lee |
|  | Sarah | Littlechild |
|  | Adam | McGeoch |
|  | Suzanne | Miller |
|  | Agnieska | Osmanska |
| **North Middlesex Hospital** | John | Bridgewater (PI) |
|  | Ernesto | Balaguer-Ruiz |
|  | Girish | Bhome |
|  | Moira | Durdy |
|  | Lorraine | Hurl |
|  | Shardul | Kulkarni |
|  | Simranjit Kaur | Mehta |
|  | Lucinda | Melcher |
|  | Julia | Rees |
|  | Jamila | Roehrig |
|  | Rahi | Shah |
|  | Chloe | Van Someren |
| **Queen Alexandra Hospital** | Ann | O'Callaghan (PI) |
|  | Oluwatobi | Adeagbo |
|  | Suhail | Baluch |
|  | Kathy | Blight |
|  | Sherilee | Cook |
|  | Heather | Cuell |
|  | Tracey | Dobson |
|  | Mya | Gyi |
|  | Antony | Higginson |
|  | Samuel Luke | Hill |
|  | Chloe | Holden |
|  | Tracey | Lee |
|  | Jayne | McCartney |
|  | Badrriyya | Mohamedali |
|  | Sethupathi | Muthuramalingam |
|  | Andras | Nagy |
|  | Eleanor | Taylor |
|  | Mary | Wands |
|  | Robert | Williams |
|  | Carole | Wragg |
| **Weston General Hospital** | Stephen | Falk (PI) |
|  | Paola | Di Nardo (PI) |
|  | Marjorie | Tomlinson |
|  | Kathy | Beard |
|  | Sandra | Beech |
|  | Hannah | Berry |
|  | Debbie | Coles |
|  | Donna | Cotterill |
|  | Harvey | Dymond |
|  | Symeon | Eleftheriadis |
|  | Rajesh | Gamare |
|  | Christine | Graham |
|  | Serena | Hilman |
|  | Sarah | Kidd |
|  | Denise | Leighton-Price |
|  | Hugh | Lloyd-Jones |
|  | Andrew | McKendrick |
|  | Kathryn | Munday |
|  | Vivienne | Pixton |
|  | Glenn | Saunders |
|  | Ed | Sheffield |
|  | Dawn | Simmons |
|  | Axel | Walther |
|  | Rachel | Warinton |
|  | Tom | Wells |
| **Glangwili General** | Mau-Don | Phan (PI) |
|  | Samantha | Coetzee |
|  | Sonya | Goriah |
|  | Praba | Gupta |
|  | Ann | Hewins |
|  | John | Murphy |
|  | Zohra | Omar |
|  | Bryan | Phillips |
|  | Meena | Raj |
|  | Kelly | Reed |
|  | Rocio | Riba |
| **Royal Albert Edward Infirmary** | Francisca Marti | Marti (PI) |
|  | Elena | Takeuchi (PI) |
|  | Jennifer | Cannon |
|  | Kate | Chilman |
|  | Shien | Chow |
|  | Louise | Devereaux |
|  | Alison | Doran |
|  | Diane | Forrest |
|  | Karen | Moss |
|  | Monica | Patel |
|  | Angela | Power |
|  | Wendy | Stevens |
| **Sunderland Royal Hospital** | Ashraf | Azzabi (PI) |
|  | Hayley | Anderson |
|  | Rod | Beard |
|  | Jane | Cole |
|  | Michelle | Edwards |
|  | Adam | Hassani |
|  | James | Henry |
|  | Vivienne | Hullock |
|  | Stephen | Laybourne |
|  | Paula | Newton |
|  | Rachel | Pearson |
|  | Ian | Pedley |
|  | Ian | Pepley |
|  | Melanie | Robertson |
|  | Fiona | Wakinshaw |
|  | Kathryn | Wright |
| **Basingstoke and North Hampshire Hospital** | Charlotte | Rees (PI) |
|  | Louise | Beattie |
|  | Victoria | Corner |
|  | Abigail | Edwards |
|  | Adrienn | Fazekasne Fulep |
|  | Angela | Frith |
|  | Julie | Gwilt |
|  | Liz | Happle |
|  | Roger | Hudson |
|  | Andrew | Jackson |
|  | Lauriane | Kernwood |
|  | Lauriane | Kerwood |
|  | Kathryn | Leach |
|  | Emma | Magras |
|  | Asmat | Mustajab |
|  | Christina | Narh |
|  | Pennie | Porter |
|  | Arun | Selvaraju |
|  | Jackie | Smith |
|  | Claire | Williams |
| **Forth Valley Royal Hospital** | Dawn | Storey (PI) |
|  | Joanne | Blackburn |
|  | Stephanie | Brogan |
|  | Raj | Burgul |
|  | Eilidh | Henderson |
|  | Jane | Keddie |
|  | Linnet | McGeever |
|  | Kaye | McIlvar |
|  | David | McIntosh |
|  | Caroline | Mcleary |
|  | Lynn | Prentice |
|  | Annette | Riley |
|  | Joanne | Robinson |
|  | Anne | Todd |
|  | Patricia | Turner |
|  | Sally | Young |
| **Mount Vernon Hospital** | Mark | Harrison (PI) |
|  | Farhan | Ahmed |
|  | Nicola | Anyamene |
|  | Nicky | Barnes |
|  | Neel | Bhuva |
|  | Sam | Bosompem |
|  | Kari | Evans |
|  | Shiv | Gayadeen |
|  | Rob | Glynne-Jones |
|  | Marcia | Hall |
|  | Rakhi | Jain |
|  | Colleen | Murray |
|  | Julie | Russell |
|  | Waqar | Saleem |
|  | Anand | Sharma |
|  | Margaret | Stone |
|  | Harsha | Vara |
| **Queen Elizabeth Hospital (Birmingham)** | Gary | Middleton (PI) |
|  | Sabia | Akhtar |
|  | Amisha | Desai |
|  | Colm | Forde |
|  | Kam | Gareja |
|  | Sharon | Hackett |
|  | Sam | Hopkins (nee Poole) |
|  | Mary | Kotadia |
|  | Victoria | Kunene |
|  | Catherine | Prest |
|  | Helen | Preston |
|  | Donna | Smith |
|  | Phillipe | Taniere |
| **Queen's Hospital Burton** | Manjusha | Keni (PI) |
|  | Ann | Adams |
|  | Mosan | Ashraf |
|  | Jo | Burns |
|  | Helen | Cox |
|  | Katy | English |
|  | Annette | Fleet |
|  | Sarah | Hathaway-Lees |
|  | Elizabeth | Kemp |
|  | Hayley | Lewis |
|  | Clare | Mewies |
|  | Jennifer | Moyes |
|  | James | Price |
|  | Scott | Sanders |
|  | Adrian | Smith |
|  | Alison | Tilley |
| **Russells Hall Hospital** | Ankit | Jain (PI) |
|  | Simon | Grumett (PI) |
|  | Joann | Atkinson |
|  | Daniel | Bull |
|  | Donna | Cleal |
|  | Lesley | Edwards |
|  | Kath | Harrow |
|  | Stacey | Jennings |
|  | Lucy | Kadiki |
|  | Karen | Kanyi |
|  | Sally | Keates-Porter |
|  | Pek | Keng-Koh |
|  | Margaret | Marriott |
|  | Julie | Matthews |
|  | Karen | McGarry |
|  | Vanessa | Moore |
|  | Andrew | Moores |
|  | Manesh | Patel |
|  | Veena | Shinde |
|  | Lucie | Smith |
|  | Lucy | Smith |
|  | Angela | Watts |
| **Singleton Hospital** | Sarah | Gwynne (PI) |
|  | Cristina | Lopez (PI) |
|  | Alya | Al-Affan |
|  | Philip | Bryant |
|  | Karen | Chesters |
|  | Sharon | Davies |
|  | Jenna | Edwards |
|  | Stuart | Evans |
|  | Tracey | Ford |
|  | Ricky | Frazer |
|  | Judith | Gooding |
|  | Olivia | Hatcher |
|  | Gillian | Jones |
|  | Lewis | Jones |
|  | Maung | Moe |
|  | Karen | Phillips |
|  | Euan | Pratt |
|  | Alex | Richards |
|  | Louise | Thomas |
|  | Julie | Turner |
|  | Nia | Viney |
|  | Dawn | Withers |
| **University Hospital Coventry** | Vanessa | Potter (PI) |
|  | Jason | Allen |
|  | Senthil Kumar | Athmanathan |
|  | Rachel | Bazeley |
|  | Susan | Bird |
|  | Yasmin | Brough |
|  | Maggie | Brown |
|  | Dannielle | Burgess |
|  | Luanne | Carey |
|  | Philippa | Clark |
|  | Peter | Correa |
|  | Kishore | Gopalakrishnan |
|  | Cheryl | Hunter |
|  | Sian | Kempster |
|  | Mohammed | Khan |
|  | Fiona | McGurk |
|  | Jade | McKelvie |
|  | Lucy | Miller |
|  | Sarah | O'Toole |
|  | Karandeepu | Pachoo |
|  | Noor | Shaw |
|  | Laura | Stanley |
|  | Charlie-marie | Suddens |
|  | Rachel | Thompson |
|  | Maria | Truslove |
|  | Linda | Wimbush |
|  | Jane | Wording |
| **University Hospital of North Tees** | Madhavi | Adusumalli (PI) |
|  | David | Wilson (PI) |
|  | Alison | Chilvers |
|  | Helen | Dunn |
|  | Sarah | Essex |
|  | Mohammad | Hegab |
|  | Hyder | Latif |
|  | Moira | Percival |
|  | Sarah | Pitcairn |
|  | Lynda | Poole |
|  | Pam | Race |
|  | Andrew | Sigsworth |
|  | Eleni Andriana | Trigka |
|  | Helen | Wardle |
|  | Bill | Wetherill |
| **Whittington Hospital (London)** | Pauline | Leonard (PI) |
|  | Rashidat | Adeniba |
|  | Dhili | Arul |
|  | Jonathan | Flor |
|  | Kavita | Kantilal |
|  | Xiao Lou | Lu |
|  | Mulyati | Mohamed |
|  | Michelle | Saull |
|  | Nuray | Temiz |
|  | Azmina | Verjee |
|  | Simon | Wan |
| **Freeman Hospital, Newcastle** | Ashraf | Azzabi (PI) |
|  | Craig | Alderson |
|  | Chris | Barron |
|  | Michelle | Borthwick |
|  | Julie | Burton |
|  | Kay | Carson |
|  | Fiona | Chapman |
|  | Sarah | Cook |
|  | Fareeda | Coxon |
|  | Sue | Farrell |
|  | Elaine | Greaves |
|  | Ahmed | Hashmi |
|  | Amanda | Henderson |
|  | Kathryn | Hewitt |
|  | Ben | Hood |
|  | Thomas | Jarvis |
|  | Irene | Jobson |
|  | Najibah | Mahtab |
|  | Lesley | Naik |
|  | Stephanie | Needham |
|  | Gemma | O'Neill |
|  | Ian | Pedley |
|  | Sindhu | Ramamurthy |
|  | Zarine | Razvi |
|  | Elizabeth | Reay |
|  | Timothy | Simmons |
|  | Carole | Stobbart |
|  | Jonathan | Stoddart |
|  | Nichola | Waugh |
|  | Hesther | Wilson |
| **Leighton Hospital** | Michael | Braun (PI) |
|  | Vanessa | Adamson |
|  | Carole | Bennion |
|  | Kim | Best |
|  | Leanne | Everall |
|  | Julia | Gemmell |
|  | Laura | Hanton |
|  | Christy | Henderson |
|  | Adele | Hough |
|  | Chris | Hough |
|  | Cyndy | Jackson |
|  | Taya | Jones |
|  | Tracy | Larcombe |
|  | Carolyn | Mansfield |
|  | Emma | Margerum |
|  | Julie | Meir |
|  | Andrew | Ritchings |
|  | Paul | Simcock |
|  | Sarah | Tinsley |
|  | Caroline | Walker |
| **Ninewells Hospital, Dundee** | Sharon | Armstrong (PI) |
|  | Jennifer | Allison |
|  | Rachael | Banks |
|  | Anne | Black |
|  | Louise | Brannan |
|  | Frank | Carey |
|  | Shona | Carson |
|  | Helen | Cumming |
|  | Debbie | Forbes |
|  | Audrey | Lyall |
|  | AJ | Munro |
|  | Moira | Rogers |
|  | Ian | Sanders |
|  | Gail | Weir |
| **Westmorland General Hospital** | David | Eaton (PI) |
|  | Rebecca | Anderson |
|  | Syed | Asghar |
|  | Manal | Atwan |
|  | Claire | Bartlett |
|  | Ashoke | Biswas |
|  | Jennifer | Bowler |
|  | Karen | Burns |
|  | Rebecca | Calvert |
|  | Amy | Ford |
|  | Laura | Healey |
|  | Nima | Herlekar |
|  | Maria | Kassi |
|  | Lauren | Kilifin |
|  | Jo | Kilkenny |
|  | Nicola | Mackenzie |
|  | Aileen | Menzies |
|  | Helen | Morris |
|  | Debbie | Power |
|  | Jane | Ritchie |
|  | Mary | Robinson |
|  | Vickie | Rose |
|  | Rachel | Simmons |
|  | Andrew | Taylor |
|  | Hilary | Thatcher |
|  | Gail | Wiley |
| **Belfast City Hospital** | Victoria | Coyle (PI) |
|  | Conal | Askin |
|  | Ellen | Brown |
|  | Karen | Campfield |
|  | Catherine | Davidson |
|  | Michael | Hanna |
|  | Diane | Law |
|  | Alison | McKeever |
|  | Aine | McKeown |
|  | Damian | McManus |
|  | Linda | McNeice |
|  | Karen | Parsons |
|  | Miranda | Reid |
|  | Fiona | Tarpey |
|  | Joanne | Todd |
|  | Paul | Ward |
|  | Richard | Wilson |
| **Dorset County Hospital** | Amelie | Harle (PI) |
|  | Richard | Osborne (PI) |
|  | Pauline | Ashcroft |
|  | Corrado | d'Arrigo |
|  | Maxine | Flubacher |
|  | Jackie | Gibbins |
|  | Karen | Hogben |
|  | Arabis | Oglesby |
|  | Andrew | Rees |
|  | Simon | Wilsher |
| **Great Western Hospital** | Sarah | Lowndes (PI) |
|  | Graham | Brown |
|  | Christopher | Clarke |
|  | Amanda | Colston |
|  | Jan | Dodge |
|  | Eva | Fraile |
|  | Sarah | Grayland |
|  | Lesley | Haxton |
|  | Lawrence | John |
|  | Jean | Kordula |
|  | Lynsey | Kyeremeh |
|  | Donna | Lake |
|  | Catherine | Lewis Clarke |
|  | Sarah | Long |
|  | Dorota | Marciniak |
|  | Laura | McCafferty |
|  | Darren | McFadden |
|  | Sue | Meakin |
|  | Chanelle | Meyer |
|  | Tim | Owen |
|  | Cerila | Parajes |
|  | Ronak | Patel |
|  | Suzannah | Pegler |
|  | Caroline | Pensotti |
|  | Joseph | Stevens |
| **Milton Keynes University Hospital** | Wasiru | Saka (PI) |
|  | Ann | Abraham |
|  | Hannah | Ansell |
|  | Sam | Bosompem |
|  | Matthew | Burnett |
|  | Chris | Ford |
|  | Chloe | Green |
|  | Sara | Greig |
|  | Penni | Hawkins |
|  | Chamene | Hicks |
|  | Aarzoo | Ilyas |
|  | Charity | Masvaure |
|  | Louise | Moran |
|  | Mala | Nathvani |
|  | Cheryl | Padilla-Harris |
|  | Vijay | Patel |
|  | Shahriar Mohammed | Reza |
|  | Syed Azhar Javed | Rizvi |
|  | Abby | Skillington |
|  | Jeannette | Smith |
|  | Oliver | Spring |
|  | Heather | Thomas |
|  | Stephanie | Thorp |
|  | Valerie | Webb |
|  | Dona | Wingfield |
|  | Christopher | Woodard |
| **New Cross Hospital** | Simon | Grumett (PI) |
|  | Syed | Asghar |
|  | Vanda | Carter |
|  | Sandeep | Dhillon |
|  | Anna | Grant |
|  | Clare | Hammond |
|  | Kelly | Kauldhar |
|  | Margaret | King |
|  | Christine | Kirk |
|  | Claire | Lomas |
|  | Manel | Mangalika |
|  | Gurminder | Sahota |
|  | Elaine | Wylde |
| **Pilgrim Hospital** | Zuzana | Stokes (PI) |
|  | Antoinette | Adu |
|  | Simon | Archer |
|  | Gloria | Barone |
|  | Jayne | Borley |
|  | Wendy | Deamer |
|  | Jo | Fletcher |
|  | Matthew | Flook |
|  | Amy | Kirkby |
|  | Victoria | Knight |
|  | Tara | Lawrence |
|  | Beverley | Mashegede |
|  | Helen | Palmer |
|  | Kerry | Pettitt |
|  | Gunjan | Phalod |
|  | Manuel | Ruiz-Echarri |
|  | Gemma | Sankey |
|  | Thomas | Sheehan |
|  | Rebecca | Spencer |
|  | Kinga | Szymiczek |
|  | Isobel | Thomas |
| **Rotherham District General Hospital** | Joanne | Hornbuckle (PI) |
|  | Matthew | Barnes |
|  | Sarah | Besley |
|  | Meredyth | Harris |
|  | Kath | Lowe |
|  | Scott | Nicol |
|  | Susan | Oakley |
|  | Amy | Rees |
|  | Charlotte | Widdop |
| **Royal Bournemouth Hospital** | Tamas | Hickish (PI) |
|  | Jocelyn | Ablorde |
|  | Omolade | Bakarey |
|  | Rachel | Bower |
|  | Zoe | Clark |
|  | Nicole | Davies |
|  | Alison | Hogan |
|  | Stephanie | Jones |
|  | Tiffany | Joyce |
|  | Maria | Lane |
|  | Sharon | Megson |
|  | Sandy | Pressdee |
|  | Linda | Purandare |
|  | Taslima | Rabbi |
|  | Emma | Sharland |
|  | Esther | Una Cidon |
|  | Luke | Vamplew |
|  | Jasmin | Webb |
| **Royal Marsden Hospital (London)** | Ian | Chau (PI) |
|  | Helen | Breeze |
|  | Shirley | Clifton |
|  | Saoirse | Dolly |
|  | Sandra | Esteban Moreno |
|  | Lucy | Featherstone |
|  | Shelby | Hatt |
|  | Blanka | Hezelova |
|  | Alexander | Lee |
|  | Hazel | Lote |
|  | Lizzie | Love |
|  | Nnenna | Ngwu |
|  | Isma | Rana |
|  | Gihan | Ratnayake |
|  | Penny | Rogers |
|  | Clare | Saffery |
|  | Anna | Scott |
|  | Izelle | Ueckermann |
|  | Chloe | Westrip |
|  | Ian | Chau |
|  | Sally | Abdelmalik |
|  | Gayahri | Anandappa |
|  | Joo Ern | Ang |
|  | Thushasa | Ansari |
|  | Sheila | Azaiji-Benjamin |
|  | Annette | Bryant |
|  | Shirley | Clifton |
|  | Richard | Crux |
|  | David | Cunningham |
|  | Sara | Diffley |
|  | Julie | Duncan |
|  | Laurice | Edwards |
|  | Sandra | Esteban Moreno |
|  | Lucy | Featherstone |
|  | Monika | Ferencova |
|  | Angela | Gillbanks |
|  | Sarnjeet | Kaur |
|  | Naila | Kaudeer |
|  | Shelize | Khakoo |
|  | Shannon | Kidd |
|  | Retchel | Lazaro Alcausi |
|  | Hazel | Lote |
|  | Jacqueline | Oates |
|  | Bijal | Patel |
|  | Minal | Patel |
|  | Brenda | Pem |
|  | Sijy | Pillai |
|  | Clare | Saffery |
|  | Francesco | Sclafani |
|  | Gillian | Smith |
|  | Eleanor | Temple |
|  | Jan | Thomas |
|  | Andrea | Turner |
|  | Izelle | Ueckermann |
|  | David | Watkins |
