# Supplementary material for: Experiences of running a stratified medicine adaptive platform trial: Challenges and lessons learned from 10 years of the FOCUS4 trial in metastatic colorectal cancer
Source: Clin Trials. 2022 Jan 27;19(2):146–57. doi: 10.1177/17407745211069879 (PMC9036145; doi:10.1177/17407745211069879)
Supplement: sj-pdf-2-ctj-10.1177_17407745211069879 – Supplemental material for Experiences of running a stratified medicine adaptive platform trial: Challenges and lessons learned from 10 years of the FOCUS4 trial in metastatic colorectal cancer [file sj-pdf-2-ctj-10.1177_17407745211069879.pdf]

FOCUS4 was launched in 2014 and completed recruitment in 2020. We are currently submitting abstracts to ASCO 2021 and drafting publications. You should have received a link to our closed investigators meeting on 5th March to hear the results of arms N and C.

We are very keen to include your feedback on the trial in an overarching paper on the FOCUS 4 Platform and plan to include all contributors as authors given the huge team effort across the UK – so tell us the good or bad - and also share any lessons learned in the short questionnaire below;

The survey takes 6 minutes and is completely anonymous.

We know all staff are really busy at the moment so we have kept the survey really short. **We are particularly interested in the free text boxes**.

Please do forward this link to other colleagues (e.g. trials nurses, pharmacy, pathologists, CTCs, R&D departments or patient/carer representatives) but do note the tight turn around to **complete the survey by Friday 19th February**. 0

Question Title

* 1. What level site are you from? 0

- ☐ Level 1
- ☐ Level 2
- ☐ Level 3
- ☐ Not sure

ﬂ

Question Title

* 2. What was your role on FOCUS4? 0

- ☐ Site PI
- ☐ Radiologist
- ☐ Other clinical
- ☐ Research nurse
- ☐ Research trial coordinator
- ☐ Pathologist
- ☐ Pathology lab staff
- ☐ Pharmacist
- ☐ R&D
- ☐ Patient/carer representatives
- ☐ Other (please specify)

[ ]

NEW QUESTION

Question Title

* 3. Your standard of care for patients with unresectable stage four disease 0

	None of the time	Some of the time	Most of the time	All of the time	N/A
Chemo till progression	○ Chemo till progression None of the time	○ Chemo till progression Some of the time	○ Chemo till progression Most of the time	○ Chemo till progression All of the time	○ Chemo till progression N/A

	None of the time	Some of the time	Most of the time	All of the time	N/A
Intermittent with a complete break	Intermittent with a complete break None of the time	Intermittent with a complete break Some of the time	Intermittent with a complete break Most of the time	Intermittent with a complete break All of the time	Intermittent with a complete break N/A
Intermittent chemo with maintainance 5fu/ cap	Intermittent chemo with maintainance 5fu/ cap None of the time	Intermittent chemo with maintainance 5fu/ cap Some of the time	Intermittent chemo with maintainance 5fu/ cap Most of the time	Intermittent chemo with maintainance 5fu/ cap All of the time	Intermittent chemo with maintainance 5fu/ cap N/A
Chemo for a set duration e.g. 24 weeks	Chemo for a set duration e.g. 24 weeks None of the time	Chemo for a set duration e.g. 24 weeks Some of the time	Chemo for a set duration e.g. 24 weeks Most of the time	Chemo for a set duration e.g. 24 weeks All of the time	Chemo for a set duration e.g. 24 weeks N/A

Question Title

* 4. Has taking part in FOCUS4 altered this? 0

- Yes
- Not yet, but it might
- No
- None of the above

Any comments? Please enter them here.

Question Title

* 5. To what extent do you agree with the following statement: The inability to restart EGFRI after a break impacted on who I selected for the trial. 0

Strongly Disagree	Disagree	Neither Agree nor Disagree	Agree	Strongly Agree
Strongly Disagree	Disagree	Neither Agree nor Disagree	Agree	Strongly Agree

If so, how?

Question Title

* 6. To what extent do you agree with the following statement: Having an unselected substudy (FOCUS4-N) was an important element. 0

Strongly Disagree	Disagree	Neither agree nor disagree	Agree	Strongly Agree
Strongly Disagree	Disagree	Neither agree nor disagree	Agree	Strongly Agree

Any comments? Please enter them here.

Question Title

7. What were the advantages/disadvantages of performing a trial in the maintenance setting? 0

Question Title

* 8. Because we were investigating different molecular cohorts where there might be prognostic differences we thought It was important to include a control arm.
Did you think this made the trial more difficult? 0

Yes

No

Any comments? Please enter them here.

Question Title

9. The TMG worked hard with frequent disappointment to obtain novel agents for the trial. Do you have any concrete suggestions about how this could be done better in future? 0

Question Title

10. Can you suggest a more novel or exciting hypothesis for an unselected arm? 0

There are so many things you could tell us about: Usability of the protocol, interest in the arms, comprehensiveness of the biomarker panel and the tissue testing process, ability to RECIST report, having 3 site levels, electronic data capture, CTU interaction and engagement with local R&D or network support but feel free to raise anything you would like. 0

Question Title

11. Did you face any particular challenges with the trial? 0

Question Title

12. What went well in FOCUS4?
This could include comments on the design or trial management. 0

Question Title

13. In terms of future studies in molecular cohorts do you feel that platform trials are the best way to proceed? If so, is there anything that would add to a recipe for future success? 0

Question Title
14. Finally is there anything else you would like to see included in a publication to inform the community that we haven't covered? 0

NEW QUESTION
There are so many things you could tell us about and below are some examples:

Usability of the protocol, interest in the arms, comprehensiveness of the biomarker panel and the tissue testing process, ability to RECIST report, having 3 site levels, electronic data capture, CTU interaction and engagement with local R&D or network support but feel free to raise anything you would like. 0

Question Title
15. What were the biggest challenges to recruitment at your site? 0

Question Title
16. What went well in FOCUS4? 0

Question Title
17. To what extent do you agree with the following statement:
The tissue testing process was straightforward 0

Strongly disagree	Disagree	Neither agree nor disagree	Agree	Strongly agree	N/A
Strongly disagree	Disagree	Neither agree nor disagree	Agree	Strongly agree	N/A

Any comments about the tissue testing process? Please enter them here.

Question Title
18. We used MACRO as the Electronic Data Capture system. We appreciate from sites there were challenges with this. What works well or is particularly difficult with eDCs?
(if applicable) 0

Question Title
19. What can we do better in the next stratified medicine study in colorectal cancer? 0

Question Title

20. Any thoughts for future studies? Are platform trials the way to go? If so, is there anything that would add to a recipe for future success? 0

Question Title

21. Is there anything else you would like to see included in a publication to inform the community that we haven’t covered? 0

Question Title

22. Feel free to add any other comments here. 0
